# Impact of Titanium Dioxide on Water Uptake and Diffusion in Nafion

**DOI:** 10.1021/acs.jpcc.5c00822

**Published:** 2025-05-19

**Authors:** Madeline Garell, Natechanok Yutthasaksunthorn, Hakhyeon Song, Johannes Leisen, Marta C. Hatzell

**Affiliations:** † George W. Woodruff School of Mechanical Engineering, 1372 Georgia Institute of Technology, Atlanta, Georgia 30332, United States; ‡ School of Chemical and Biomolecular Engineering, 1372 Georgia Institute of Technology, Atlanta, Georgia 30332, United States; § School of Chemistry and Biochemistry, 1372 Georgia Institute of Technology, Atlanta, Georgia 30332, United States

## Abstract

Inorganic fillers are commonly integrated into ion-exchange membranes to alter water hydration, improve ion conductivity, and tune transport properties. Here, we measure water uptake and diffusion in Nafion, which contains various concentrations of TiO_2_ (0–5 wt %). With adsorption–desorption isotherms measured by dynamic vapor sorption, we probe the moisture kinetics and find a maximum change in hydrated Nafion mass by water vapor adsorption at 2 wt % TiO_2_. Pulsed field gradient nuclear magnetic resonance (NMR) reveals that with increasing TiO_2_ concentration, the two measured components of water diffusion are also maximized at 2 wt % TiO_2_. The first component had a diffusion coefficient, on the order of 1 × 10^–9^ m^2^/s, which approaches the diffusion of free water. Observing root-mean-square displacement as a function of increasing observation time indicates that this water population is affected by the ionomer-TiO_2_ morphology. The second component had a diffusion coefficient on the order of 1× 10^–11^ m^2^/s, which is attributed to water molecules that interact more strongly with sulfonic acid groups. With solid-state proton NMR relaxometry under low (near 0%) and high (near 100%) relative humidity, we measure T_1_ and T_2_ relaxation of water molecules on different time scales. Hydrated Nafion+2 wt % titania offers the highest water relaxation times with T_1_ = 413 ms and T_2_ = 4.77 ms. This high T_1_/T_2_ ratio further indicates a two-component water environment and semirestricted water diffusion.

## Graphical abstract

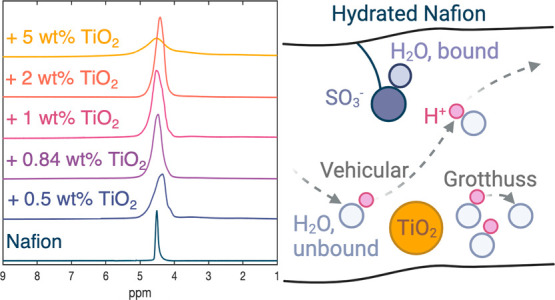

## Introduction

The growing demand for efficient energy conversion technologies underscores the importance of developing high-performance ion exchange polymers.^1^ Optimizing water transport in ionomers requires an understanding of hydration, water diffusion, and water–ionomer interactions. Despite advances in this area, key questions remain about the sorption thermodynamics, transport mechanisms, and water diffusion properties in nonporous ionomers.^2–4^ Nafion is a negatively charged perfluorosulfonic acid (PFSA) ion-conducting polymer widely used in the catalyst layer of hydrogen electrolyzers and polymer electrolyte fuel cells (PEFCs).^5–9^ Nafion is one of the best-performing polymer membrane materials in terms of proton conductivity and durability, and much recent research has aimed at overcoming limitations of the material by creating hybrid composite Nafion-based ionomers.^10^ These composite ionomers include Nafion mixed with inorganic fillers, organic fillers, or other polymers.^11,12^ Composite Nafion ionomers have demonstrated improved water sorption capacity, which is essential to achieve high proton conductivity,^13,14^ and to enable operation under favorable fuel cell conditions (high temperature and low humidity).^7,15^ Inorganic fillers that contain surface hydroxyl groups, such as TiO_2_,^7,16,17^ SiO_2_ ^17–20^ and ZrO_2_,^17^ improve ionomer water retention (Table S1).

Water transport in Nafion is highly dependent on the degree of ionomer hydration. The Nafion ionomer is generally understood to consist of a hydrophobic polytetrafluoroethylene (PTFE) backbone chain with hydrophilic sulfonic acid (−SO_3_H) side chains that facilitate water transport.^5,21^ The accumulation of water around the sulfonic acid groups widens the proton-conducting channels, allowing for high proton conductivity up to 0.1 S/cm^2^.^9^ Water diffusion has been measured in hydrated Nafion ionomers by several methods, including electrical conductivity,^8^ quasi-elastic neutron scattering (QENS),^22^ dynamic vapor sorption,^23^ static permeation,^24^ and nuclear magnetic resonance (NMR)^14^ (Table S2). There is a wide range of translational diffusion coefficients reported in the literature on Nafion because there are multiple diffusion mechanisms that occur simultaneously in the hydrated ionomer. The measurement of multiple water diffusion coefficients in Nafion is due to the populations of water molecules diffusing at different rates due to their proximity to the sulfonic acid groups.^25^ There remains a need to measure changes in the multimodal nature of water diffusion in these ionomers in the presence of inorganic fillers (e.g., TiO_2_).^26,27^ Typical water uptake in hydrated Nafion ionomers without the presence of an inorganic filler reaches 20%.^28^ Introducing 1 wt % of TiO_2_ to Nafion has proven to increase water uptake by up to 70%^15^ due to the hygroscopic nature of TiO_2_. Despite frequent use, there remains a limited quantitative understanding of how TiO_2_ affects the distribution, confinement, and diffusion of water within Nafion at the molecular level across a range of filler concentrations. Here, a combination of dynamic vapor sorption, NMR relaxation, and diffusion measurements resolves the multimodal water dynamics in Nafion–TiO_2_ composites, bridging the gap between macroscopic hydration trends and microscale diffusional transport.

Beyond the varying modes of water transport, water transport occurs differently under different environmental conditions (e.g., relative humidity). At low humidity levels, protons are known to move via the SO_3_H groups at the surface of the PTFE backbone. This is referred to as the surface mechanism (Figure 1).^9,21^ With increasing humidity, clusters of water molecules begin to form at the sulfonic acid groups enabling transport via the proton-diffusion-based vehicular mechanism as the widening of hydrophilic domains and the presence of more free water molecules results in the formation of hydronium ions diffusing in the channels.^9^ At high humidity levels, protons diffuse by proton-transfer reactions across the network of water molecules present in the proton-conducting channels. This dominant proton “hopping” is termed the Grotthuss mechanism.^29–32^ At high water content, the membrane can be considered an interconnected network of polymer rods^33^ where proton transport resistance is low, as the Nafion structure consists of connected clusters of hydrogen ions. This is in contrast to the dry ionomer morphology where isolated hydrogen ion clusters are observed by nanoscale small-angle X-ray scattering.^34,35^ The different components of water can be identified by the temperature at which the water freezes, often measured by differential scanning calorimetry experiments.^36^ Water bound to sulfonic acid groups is called nonfreezable, as molecules are strongly polarized and unable to crystallize, while water that behaves similarly to bulk water is freezable.^37^ Although increased hydration is known to enhance water diffusivity in Nafion, and multiple water populations have been previously observed, the detailed influence of inorganic fillers on the evolution of each water population has remained insufficiently characterized. This study quantifies the impact of TiO_2_ content on distinct water diffusion components and correlates these findings with water confinement and relaxation behavior, enabling a more complete understanding of the structure–transport relationships in composite ionomers.

**Figure 1:**
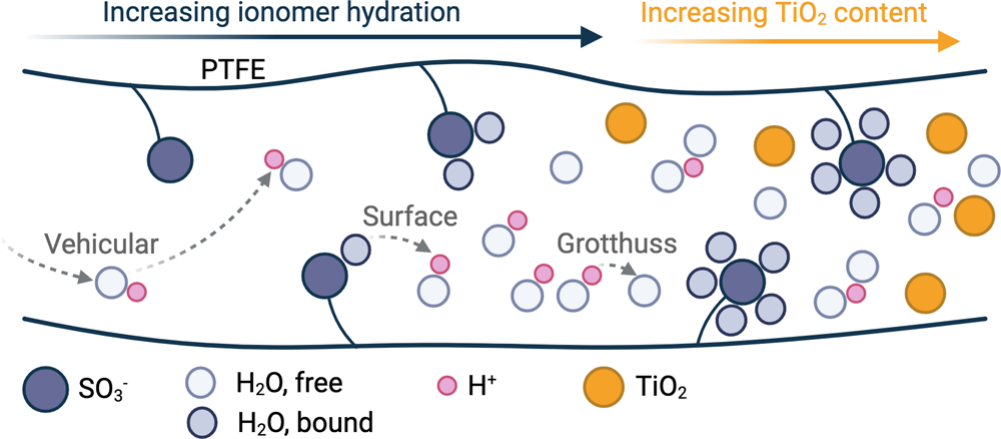
Proton transport mechanisms with increasing ionomer hydration and TiO_2_ concentration in a Nafion ionomer.

## Experimental Methods

### Nafion Sample Preparation

Activated Nafion NR50 (Millipore Sigma) beads were dissolved in N,N-dimethylformamide (DMF). Titanium­(IV) oxide nanopowder (21 nm primary particle size, Sigma-Aldrich) was added to Nafion-DMF solutions to prepare ionomer samples with 0, 0.5, 0.84, 1, 2, and 5 wt % TiO_2_. Ionomer samples were cast in glass Petri dishes (100 × 15 mm) and then placed in an oven at 60 °C until DMF evaporated and a solid Nafion–TiO_2_ film formed. Nafion samples were lifted from the glass after swelling with DI water.

### Water Uptake

To determine the initial dry ionomer weight, *W*_*d*_, Nafion samples were dried in an oven at 60 °C for 24 h and then immediately weighed. To measure the fully hydrated weight, *W*_*h*_, dry ionomer samples were immersed in DI water for 48 h, blotted dry with weighing paper, and then immediately weighed. Water uptake was calculated according to the following equation:

$$\mathrm{Water}\mathrm{uptake}\left(\%\right)=\frac{W_{h}-W_{d}}{W_{d}}\times 100$$ 1

### Differential Scanning Calorimetry

Differential scanning calorimetry (DSC) experiments were carried out on a DSC 250 instrument (TA Instruments). Samples (3–5 mg) were equilibrated in DI water for 24 h before being sealed in Tzero pans with hermetic lids. The DSC program first equilibrated the sample at −50 °C, then ramped the temperature from −50 to 250 °C at a rate of 10 C/min. The experiments were performed under a nitrogen atmosphere using a flux of 50 mL/min. The mass of freezeable water was calculated by integrating the endothermic peak near 0 °C (enthalpy of freezing water = 314 J/g), and the total mass of water was calculated by integrating the endothermic peak near 150 °C (enthalpy of water vaporization = 2258 J/g).^38^

### Dynamic Vapor Sorption

Adsorption and desorption isotherms at 30 °C were recorded by using an IgaSorp analyzer. Hydrated Nafion samples were dried at 70 °C for 2 h. The temperature was then reduced to 30 °C for the adsorption–desorption experiment. Relative humidity was increased in steps from 0 to 95% and then reduced back to 0%, ensuring that the sample reached equilibrium before each step change in humidity (Figure S1). Equilibrium criteria were set as 0.003 wt % change in 1 min. Data was recorded every 2 min or for every 0.01 wt % change in sample mass.

The Zimm–Lundberg (Z–L) cluster function^39^ provides information on the clustering of sorbed molecules in polymer systems as measured by dynamic vapor sorption measurements. The Z–L function is dependent on water volume fraction, ϕ_*w*_, and water activity, *a*_*w*_,

$$\frac{G_{s}}{V_{w}}=-\left(1-\phi_{w}\right){\left[\frac{\delta \left(\frac{a_{w}}{\phi_{w}}\right)}{\delta a_{w}}\right]}_{p,T}-1$$ 2

where *G*_*s*_ is the cluster integral and *V*_*w*_ is the partial molecular volume of water. If the value of the cluster function (*G*_*w*_/*V*_*w*_) is greater than −1, that indicates that the sorbed molecules are forming clusters. The average number of molecules within a cluster is evaluated from the following equation: where MCS is the mean cluster size.

$$\mathrm{MCS}=1+\left[\frac{G_{s}\phi_{w}}{V_{w}}\right]$$ 3

Nafion exhibits a sigmoidal water vapor adsorption isotherm, known as a type II isotherm. This type of isotherm is concave to the *x*-axis at low water activity and convex at high water activity. The shape of the isotherm is a combination of Langmuir and Flory–Huggins isotherms.^40^ There are several models commonly used to fit adsorption isotherm data, including the BET type II equation, the Guggenheim-Anderson-de Boer model, or the Park equation.^41,42^ Here, we employ the new dual-mode sorption (DMS) model based on multilayer adsorption theory to model water adsorption in Nafion with TiO_2_.^43^

$$c=C_{p}\frac{k^{\prime }a}{1-k^{\prime }a}+C_{p}\frac{\left(A^{\prime }-1\right)k^{\prime }a}{1+\left(A^{\prime }-1\right)k^{\prime }a}$$ 4

Here, *c* is the sorbate concentration in the ionomer and *a* is the water activity. The DMS model has three parameters that describe the adsorption. *C*_*p*_ is the weighted mean value of the sorption capacity of the ionomer relative to water vapor. This parameter depends on the state and structure of the polymer and is related to the mean number of water molecules sorbed on Langmuir sites. *A*′ is a measure of the difference between the interactions of the first layer of vapor molecules with the microvoid and the interactions between successive layers of vapor molecules with the microvoid. And *k′* is a measure of the interaction between the ionomer material and water vapor. The DMS model describes the two regions of water that form within Nafion as the ionomer is hydrated. The first shell of strongly adsorbed water in the ionomer microvoids is modeled with the second term of the equation ( $c_{2}=C_{p}\frac{k^{\prime }a}{1-k^{\prime }a}$ ) and subsequent layers of weakly adsorbed water in the ionomer matrix are described by the first term ( $c_{1}=C_{p}\frac{\left(A^{\prime }-1\right)k^{\prime }a}{1+\left(A^{\prime }-1\right)k^{\prime }a}$ ).

Diffusion coefficients were calculated from adsorption isotherms according to the following equation

$$\frac{M_{t}}{M_{\infty }}=\frac{4}{d}\sqrt{\frac{Dt}{\pi }}$$ 5

where *M*_*t*_ is the amount adsorbed at time *t*, for a given relative humidity, *M*_*∞*_ is the amount adsorbed at thermodynamic equilibrium, *D* is the diffusion coefficient of the adsorbate, and *d* is the adsorbent thickness.^23^ To measure the film thickness, we use cross-section scanning electron microscopy (SEM). Nafion films were hydrated to 50% relative humidity using a saturated Mg­(NO_3_)_2_ solution.^44^ The solution-cast Nafion ionomers were cracked using liquid nitrogen and then coated with gold nanoparticles using a Hummer sputtering system. Cross-sectional SEM was performed on a Hitachi SU 8230 microscope. Film thicknesses were measured by using ImageJ.

### Pulsed Field Gradient NMR

^1^H diffusion NMR experiments were performed on a Bruker AVIII 400 MHz spectrometer equipped with a dedicated diffusion probe. After being blotted dry, a hydrated ionomer sample was placed in a 5 mm NMR tube and capped with a piece of cotton soaked in DI water to maintain high relative humidity throughout the experiments. Hydrated samples were prepared and measured on the same day to ensure no change in the ionomer hydration. Gradient pulse duration was 1 ms, diffusion time ranged from 20 to 2000 ms, temperature was constant at 25 °C, and the number of scans equaled 32. A stimulated echo sequence was used with dwell time = 5 μs, pulse duration = 9.48 μs, and gradient strength varying from 5 to 250 G/cm. To mitigate artifacts, 1D reference spectra were collected to check chemical shift positions and perform baseline corrections before starting the PFG experiments. Probes were tuned prior to starting experiments, and 4 dummy scans were used to equilibrate the system before data acquisition.

The motion measured by PFG NMR is on the order of milliseconds, and water diffusion coefficients in Nafion measured by this technique range from 1× 10^–5^ to 1× 10^–15^ m^2^/s. PFG NMR is characterized by an echo sequence of radio frequency pulses and gradient pulses. A Fourier transform of the ^1^H PFG radiofrequency acquisition gives a series of spectra in which an exponential decay of peak intensity is observed for varying gradient pulse strengths. This data is characterized by the Stejskal–Tanner eq (eq 6a) to calculate the translational diffusion coefficients for protons (i.e., mobile water molecules) in the ionomer. A two-component Stejskal-Tanner equation^45^ described signal attenuation as a function of diffusion and experimental parameters.

$$I=I_{0,1}\exp\left(-D_{1}\beta \right)+I_{0,2}\exp\left(-D_{2}\beta \right)$$ 6a

$$\beta =\gamma^{2}G^{2}\delta^{2}\left(\Delta -\frac{\delta }{3}\right)$$ 6b

Diffusion coefficients were calculated from the double exponential (eq 6a), where β is a parameter that depends on the experimental conditions and properties of the nucleus under investigation (eq 6b). Here, *I* is the signal intensity, *I*_0_ is the signal intensity at zero gradient strength, γ is the gyromagnetic ratio of the probe nucleus, δ is the pulse duration, *G* is the gradient pulse strength, *D* is the translational diffusion coefficient, and Δ is the time between the two gradient pulses.^46^ In eq 6a, subscripts 1 and 2 denote the two populations of water (weakly adsorbed and strongly adsorbed) that contribute to the signal attenuation. The two-component exponential fit of signal data yields two distinct water diffusion coefficients: faster diffusion *D*_1_ on the order of 10^–9^ m^2^/s and slower diffusion *D*_2_ on the order of 10^–11^ m^2^/s. Single exponential fits (Figure S2) and double exponential fits (Figure S3) are compared to validate the use of a two-component model.

NMR is a reliable method for measuring the self-diffusion coefficients of ions in ionomers independent of the effects of interfacial transport and polymer swelling kinetics.^14,47^ NMR diffusion measurements track molecular motion at small length scales to measure local thermodynamic (self-) diffusion coefficients as well as molecular relaxation times.^48–51^ Thus, we used NMR to examine water–ionomer interactions and the modes of water transport in Nafion ionomers, which contain varying concentrations of an inorganic filler. We believe that this could have broad implications for understanding water transport in cation-exchange ionomers that contain inorganic fillers or catalysts (e.g., bipolar membranes, composite membranes, and catalyst layers within electrodes).

The degree of confinement in the ionomer for the two populations of water diffusion is understood by calculating the root-mean-square displacement of molecules with increasing experimental diffusion time, Δ.^52^

$$<x^{2}{>}^{1/2}=<x>=\sqrt{2D\Delta }$$ 7

Here, *x* is the displacement and *D* is the translational diffusion coefficient. Increasing the diffusion time and measuring the diffusion coefficients allows us to quantify information about the confinement of the diffusing molecules.^46^ Since water molecules are confined in the pores of the ionomer, increasing the experimental observation time, Δ, from 20 to 2000 ms results in a nonlinear trend in the root-mean-square displacement since molecules travel shorter distances as they encounter boundaries. At intermediate time scales, the diffusion coefficient depends on the experimental observation time and the local environment. Time-dependent diffusion measurement in solid media is often affected by both confinement and obstruction; confined particles feel the effect of the boundary with increasing diffusion time, and the presence of larger species hinders free diffusion of the observed species. Calculating the root-mean-square displacement with increasing diffusion time, Δ, demonstrates the confined nature of water molecules in Nafion when comparing it to the displacement of free molecules.

### Solid-State NMR

^1^H solid-state NMR experiments were performed on a Bruker AVIII 400 MHz spectrometer. The diffusion probe was not suitable due to its limited bandwidth, so the magic angle spinning probe was used with no spin. Nafion–TiO_2_ samples were pulverized into a fine powder, then packed in a 4 mm diameter rotor, and the ^1^H NMR spectra were acquired at 25 °C. For low relative humidity samples, the powder was dried in an oven before experiments. For high relative humidity samples, the Nafion–TiO_2_ powder was exposed in a sealed environment over a K_2_SO_4_ slurry and allowed to equilibrate with the 98% relative humidity environment over 2 days (Figure S4).

T_1_ relaxation times were measured with an inversion recovery sequence. First, a 180° pulse of 7 μs is applied, followed by a second 90° pulse of 3.5 μs. The scan delay was 5 s, and the number of scans was 8. T_1_ was calculated according to the Bloch equation:

$$I_{z}=I_{0,z}\left(1-2A\exp\left(\frac{-t}{T_{1}}\right)\right)$$ 8

where *I*_*z*_ is the magnetization vector in the *z* plane, *A* is a constant coefficient accounting for imperfections of the inversion pulse, and *t* is the time delay between pulses.

T_2_ relaxation times were measured with a Carr–Purcell–Meiboom–Gill (CPMG) sequence. First, a 90° pulse of 3.5 μs is applied, followed by a 180° pulse of 7 μs. The scan delay was 2 s, and the number of scans was 32. T_2_ relaxation is characterized by an exponential decay

$$I_{xy}=I_{0,xy}\exp\left(\frac{-t}{T_{2}}\right)$$ 9

where *I*_*xy*_ is the magnetization vector in the *xy* plane and *t* is the time between pulses.

Studying the short-range motions of water molecules by measuring proton relaxation times in Nafion is important for exploring how modifications to the ionomer environment affect water transport. Relaxation times are measured at the atomic level by solid-state NMR techniques. ^1^H solid state NMR spectroscopy is useful for observing changes in the ionomer structure due to increased hydration or the presence of an inorganic filler, as well as probing local chemical environments. Recent work has focused on the study of water dynamics in Nafion ionomers at varying levels of hydration by solid-state NMR.^53^ Findings include identifying a dependence of diffusion on hydration and the formation of hydration shells at the sulfonic acid sites in three steps. As observed with adsorption isotherm experiments here, the final step allows for bulk-like water transfer.

## Results and Discussion

We first quantified the liquid water uptake in Nafion ionomers. The addition of TiO_2_ nanoparticles does not change the liquid water uptake in the Nafion ionomer from 0 to 0.84 wt %. However, liquid water uptake increases significantly (by 2*x*) when titania concentration increases to 1–2 wt %. At 2 wt % TiO_2_ loading, the ionomer achieves maximum hydration, after which a higher TiO_2_ content begins to hinder hydration (21% water uptake in Nafion to a maximum of 44% water uptake in Nafion+2 wt % TiO_2_). It is anticipated that at higher concentrations, TiO_2_ may begin to block water transport channels. This trend in water uptake suggests that the optimal TiO_2_ concentration for hydration is 2 wt %. At 5 wt % the water uptake decreased to less than 20% (Figure 2a).

**Figure 2:**
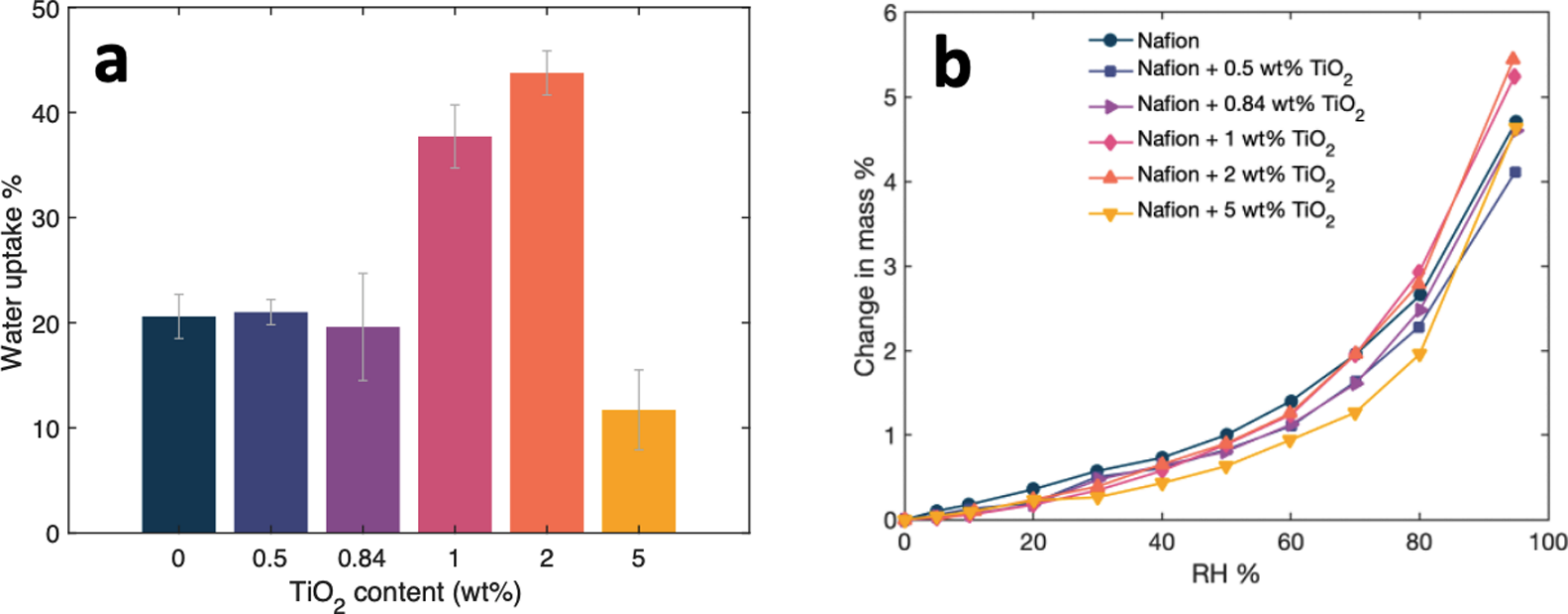
(a) Water uptake vs TiO_2_ content (error bars are the standard deviation of measurements from three ionomer samples) and (b) dynamic vapor adsorption isotherms.

Dynamic vapor sorption (DVS) experiments give insight into the adsorption and desorption kinetics of the ionomer. The sorption process in Nafion usually exhibits a sigmoidal shape characterized by three steps in the adsorption process. First, at low humidity, the sulfonic acid groups are ionized. Second, solvation shells form and begin to connect transport pathways. Third, swelling of the hydrophilic domains allows for bulk-like water diffusion. These three stages of adsorption are observed in the adsorption isotherms (Figure 2b, see Figure S5 for individual isotherms). The adsorption isotherms are fitted with the dual-mode sorption model to quantify differences in water sorption in the ionomer as the concentration of inorganic filler increases (Figure S6, fit parameters compared in Figure S7). All isotherms are well fit with the three-parameter model (adjusted *R* ^2^ > 0.99), and the value *k*′ is 0.82 for all Nafion–TiO_2_ samples, indicating that the state of the ionomer is not changing and that the difference between the water–water and water–Nafion interactions does not change. *C*_*p*_ reaches a maximum of 1.82 at 2 wt % titania (compared to 1.19 without titania), which means that at this filler concentration, there is a maximum number of water molecules strongly adsorbed at the ionized sites of the ionomer. At 2 wt % titania, *A′* reaches a minimum value of 0.60 (compared to 1.5 without titania), which means that there is a larger fraction of free water adsorbed at 2 wt % titania. As measured by DVS, the maximum mass change due to water vapor adsorption of 5.5 wt % is achieved at 2 wt % titania (compared to 4% mass change in Nafion).

The differences in water uptake by saturation in liquid water and vapor phase hydration are evident because vapor phase hydration reduces the formation of clusters of water in confined spaces in Nafion.^50^ The sigmoidal isotherm points to two populations of water adsorbed to the ionomer: one population that is strongly bound to the sulfonic acid sites and the second population that is weakly bound. The strongly bound molecules are adsorbed in a Langmuir-site first sorption shell, while the weakly bound molecules more freely distribute through the system in a second solvation shell.^40^ The adsorption–desorption isotherms (Figure S5) demonstrate the reversibility of hydration in Nafion at all inorganic filler loadings measured. The moisture uptake profile for each incremental change in humidity indicates that desorption is faster than adsorption (Figures S1 and S5). This can be explained as the ease of interconnected pores at a high water content to facilitate water removal rather than the structural reorganization required for sorption.^48^ Type II isotherms exhibit three characteristic regions that coincide with the three-step sorption process to represent a monolayer mechanism. The concavity of the isotherm at high relative humidity means that sorption in the hydrated ionomer is governed by a clustering mechanism. Evaluation of the Zimm-Lundberg function with increasing water activity supports that clustering is occurring at high RH in Nafion+TiO_2_ samples since a mean clustering size greater than 1 is observed (Figure S8). Higher water content leads to weaker interactions between the adsorbate and adsorbent, as there are stronger interactions between water molecules compared to Nafion–water interactions.

A diffusion coefficient for the adsorbing molecule can be calculated from the adsorption isotherms according to eq 5 if the thickness of the adsorbent is known. Cross-sectional SEM was used to measure the thickness of the Nafion films used in the adsorption isotherm experiments (Figure S9). The calculated water diffusion coefficient follows the trend of water uptake in the ionomer, consistent with an understanding of the hydration-dependent proton conductivity (Figure 3b). The reported values for water diffusion, measured from the adsorption isotherms of the vapor phase, are on the order of 1 × 10^–10^ m^2^/s (Table S2). The self-diffusion of free water is 2.3 × 10^–9^ m^2^/s.^54^ Diffusivities measured by DVS at 95% RH follow an exponential relationship of the form $\frac{D}{D_{0}}=a\exp\left(b\phi \right)$ where *D*_0_ is the diffusivity of water in the dry ionomer and ϕ is the water volume fraction (Figure S10). This trend indicates that there is plasticization of the ionomer (at 30 °C) and that higher water uptake and diffusion are a result of plasticization at high water activity.

**Figure 3:**
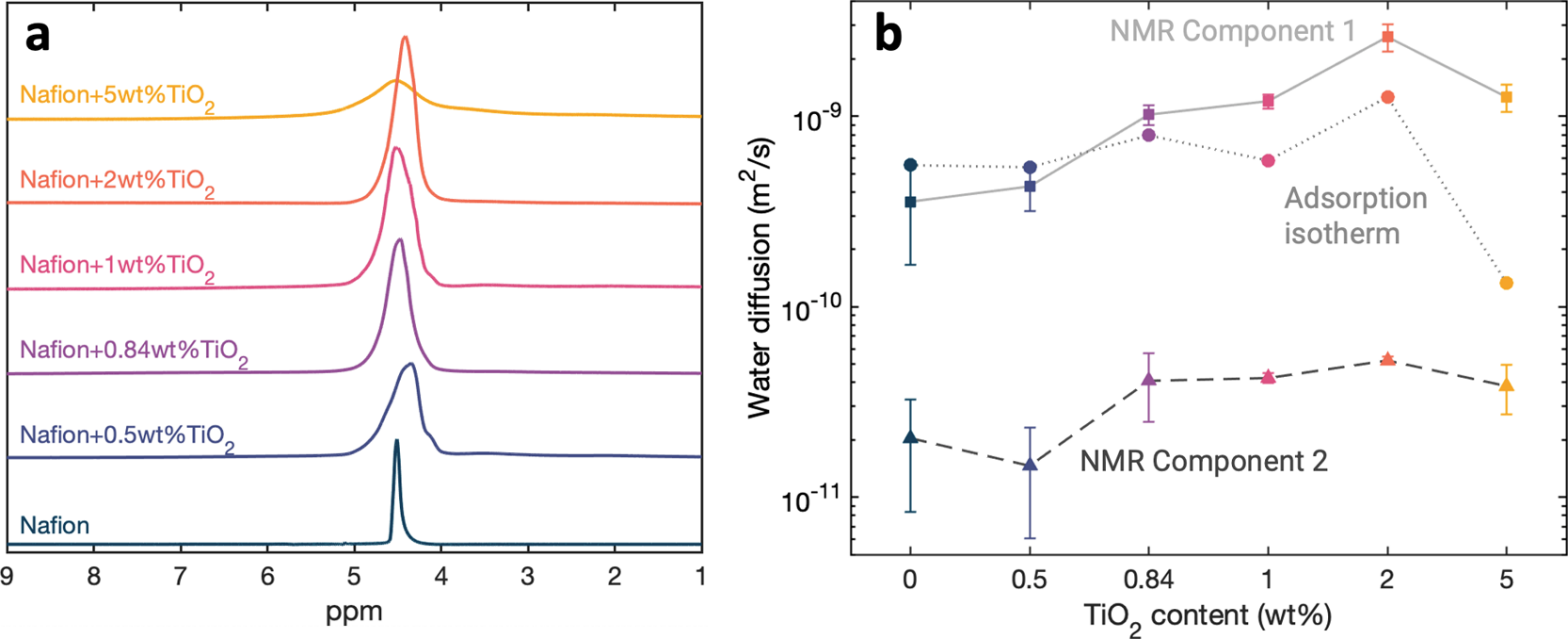
(a) ^1^H NMR spectra measured with the diffusion probe with increasing TiO_2_ content. (b) Comparison of diffusion coefficient measurements from adsorption isotherms (circle markers) and PFG NMR experiments. NMR diffusion measurements are broken down into two components, weakly adsorbed water molecule diffusion (component 1, square markers) and strongly adsorbed water molecule diffusion (component 2, triangle markers). Error bars are the standard deviations of measurements with three different Nafion–TiO_2_ samples.

A more precise view of the different mechanisms of water diffusion in Nafion is required to investigate how the concentration of TiO_2_ in the ionomer affects the different components of water diffusion. Two different water diffusion coefficients were measured in Nafion using nuclear magnetic resonance (NMR) (Table S2). With advanced characterization by NMR diffusometry and spectroscopy, we identified two distinct components of proton diffusion within a hydrated Nafion ionomer. Measurement of changes in these diffusion coefficients, which are attributed to different water transport mechanisms in Nafion, after the introduction of TiO_2_ nanoparticles is valuable for understanding the fundamental structure-transport relationships. TiO_2_, a known water dissociation catalyst, reduces the activation overpotential of water dissociation when added to the anion or cation exchange layer of a bipolar membrane. This property makes it essential to investigate the effect of TiO_2_ on water mobility within Nafion, as it is a favorable material for the cation exchange layer in bipolar membrane applications.^55^ Since ion and water diffusion depends on the degree of ionomer hydration, we expect the proton diffusion coefficients to follow the trend of water uptake, where TiO_2_ first enhances and then reduces diffusional transport.

We measure proton diffusion coefficients with ^1^H pulsed field gradient (PFG) NMR as the TiO_2_ content increases. The ^1^H spectrum (Figure 3a) of each sample provides insight into the water content, as the scale of the signal is proportional to the water content. Based on the two-component exponential model of the signal intensity, we identify one short, broad peak (strongly bound protons) and one tall, narrow peak (weakly bound protons). Both peaks in the proton spectrum, centered at 4.5 ppm, are attributed to water diffusion, since the only hydrogen nuclei in the system are water molecules and tightly bound protons at the sulfate groups. It is known that water in well-hydrated Nafion exists in two states, nonfreezing (strongly bound) and freezing (weakly bound). DSC thermograms reveal these two states of water in each of the Nafion–TiO_2_ samples, with the mass of freezeable water identified by an endothermic peak near zero (Figure S11). Across the range of titania concentrations, Nafion+2 wt % TiO_2_ exhibits the greatest amount of freezable water as well as the greatest total amount of water in the sample. The deconvolution of the NMR signal into two components corresponds to two different populations of protons in the system: strongly bound protons that are located near the Nafion SO_3_^‑^ groups and weakly bound protons that are associated with condensed water in the pores, which is transported by the Grotthuss mechanism. The fit coefficients correspond to the relative proportions of the two populations of water with different diffusion coefficients. Differences in the chemical shift of the peaks are attributed to different proton local environments. Higher chemical shift indicates lower electron density around the water molecule protons, which indicates that water is more strongly bound to the ionomer.

With pulsed field gradient (PFG) diffusion NMR, we examined the translational diffusion coefficients of the strongly adsorbed and weakly adsorbed water populations identified in the ^1^H spectra. Water self-diffusion coefficients in hydrated Nafion measured by NMR have been reported in the range of 0.5× 10^–11^–2.5 × 10^–9^ m^2^/s, showing a wide range in the diffusion of water that can be attributed to differing transport mechanisms in the ionomer (Table S2). Adding TiO_2_ to the ionomer serves to first increase the diffusion coefficients of strongly and weakly bound water molecules and then decrease the diffusion coefficients when the TiO_2_ content reaches 5 wt % (Figure 3b). Diffusion closely follows the water content in the ionomer, as a higher water content results in the transport channels becoming more interconnected rather than separated into isolated ion clusters at low water content.^8^

At the maximum hydration of 44% at 2 wt % titania, the ionomer channels expand and allow water diffusion because hydration enhances the connectivity of pathways and results in reduced tortuosity. When the TiO_2_ content reaches 5 wt %, excess filler particles obstruct these channels, inhibiting diffusion. The component 1 diffusion coefficient is reduced from 2.61× 10^–9^ m^2^/s at 2 wt % titania to 1.26× 10^–9^ m^2^/s at 5 wt % titania while the component 2 diffusion coefficient is reduced from 5.22× 10^–11^ m^2^/s at 2 wt % titania to 3.83× 10^–11^ m^2^/s at 5 wt % titania. However, even at this higher TiO_2_ concentration, the diffusion coefficients remain higher than those of pure Nafion, indicating that TiO_2_ continues to promote water transport by maintaining well-hydrated channels. Compared to pure Nafion, the component 1 diffusion coefficient is 3.5 times greater with 5 wt % titania, and the component 2 diffusion coefficient is 1.9 times greater with 5 wt % titania. Differences in measured water diffusivity arise from various testing methods at a range of time and length scales. Comparison of steady-state NMR diffusion measurements and dynamic vapor sorption diffusion measurements gives insight into the differences between diffusion and the sorption process. Lower diffusion values calculated from DVS experiments are expected, as the process involves water uptake and polymer swelling in addition to water transport. Measurement of two water populations highlights the importance of understanding how water is present in different physical states and how each population contributes to overall transport. The use of TiO_2_, a common additive in catalyst layers, provides a relevant and widely applicable model system.

Understanding the nature of the two identified transport modes is important in relating the measured diffusion coefficients to information about ionomer-water interactions. We probe the displacement of water molecules with root-mean-square displacement (RMSD) calculations. As the RMSD does not reach a plateau value as expected for a fully restricted motion, we know the observed water populations are semirestricted in Nafion (Figure 4a). Water molecules that are more strongly bound to Nafion (component 2) reach a maximum root-mean-square displacement of 8.0 × 10^–6^ m after 500 ms, while weakly interacting water molecules reach a maximum of 2.5 × 10^–5^ m after 500 ms. Water diffusing in Nafion+2 wt % TiO_2_ achieves a greater range of motion (2.5 × 10^–5^ m compared to 2.1 × 10^–5^ m in Nafion) as the ionomer is more hydrated with 2 wt % titania. Since the scale of pores in Nafion is smaller than the displacement measurements, the water molecules are semirestricted within the ionomer, with the *D*_2_ component more restricted than the *D*_1_ component. Water diffusion is hindered by the size of the channels, as well as interactions with the ionomer (Figure 4b). The slower-moving water molecules are more hindered by proximity to the ionomer SO_3_^–^ groups; thus, molecules diffusing at *D*_2_ are more restricted in motion compared to the ions at the center of the hydrated ionomer channels.

**Figure 4:**
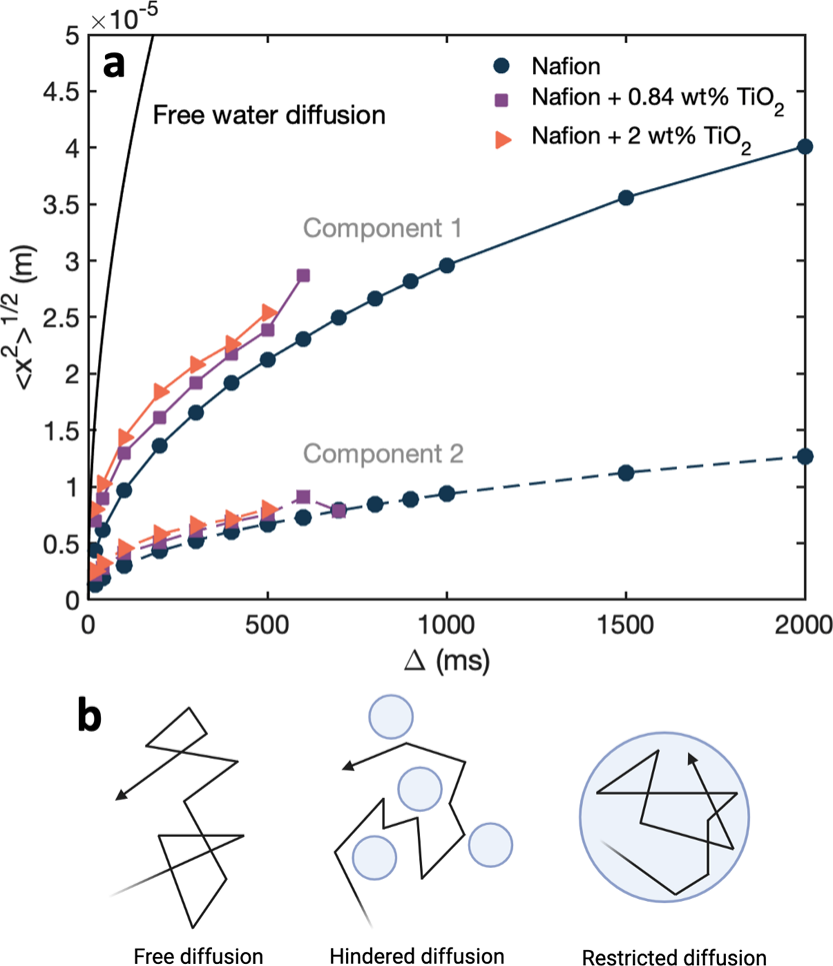
(a) Root mean square displacement vs diffusion time and (b) particle diffusion in three cases at intermediate observation time scales: free, hindered, and restricted.

From NMR experiments comparing the low relative humidity (Figure 5a) and high relative humidity (Figure 5b) Nafion ionomer ^1^H spectra, there is a narrowing of the primary peak, indicating that water is absorbed into the sample and that water mobility is increased due to the expansion of proton-conducting channels. Here, low relative humidity samples mean near-zero humidity as the Nafion has been dried; however, there is a small amount of H^+^ that remains very strongly linked by hydrogen bonds to the SO_3_^–^ groups. These water molecules are not removed even when Nafion is heated to over 200 °C.^25,56^ In the low-humidity state, there is little to no water diffusing, so the addition of any amount of TiO_2_ only serves to load the pores of the ionomer rather than exert a positive effect on water mobility. In the high (98%) humidity state, the presence of different amounts of TiO_2_ changes the mobility of water molecules in a manner similar to the trend in water uptake due to the reduced tortuosity of the ionomer morphology at high relative humidity. We can compare the ^1^H spectra measured with the diffusion probe to the spectra measured with the MAS probe for the high-humidity samples. While the sample is in the form of a film during the diffusion measurements, it has been pulverized into a fine powder for the static solid-state NMR measurements. Under high humidity, the Nafion+2 wt % titania peak has the highest intensity, while with 5 wt % titania, the intensity is reduced by 65%. Under low humidity conditions, the Nafion peak has the highest intensity, while with 5 wt % TiO_2_, the intensity is reduced to 66% of the Nafion peak intensity. For comparison between the two environments, the dry Nafion+5 wt % titania peak intensity is 35% of the hydrated Nafion+5 wt % peak intensity.

**Figure 5:**
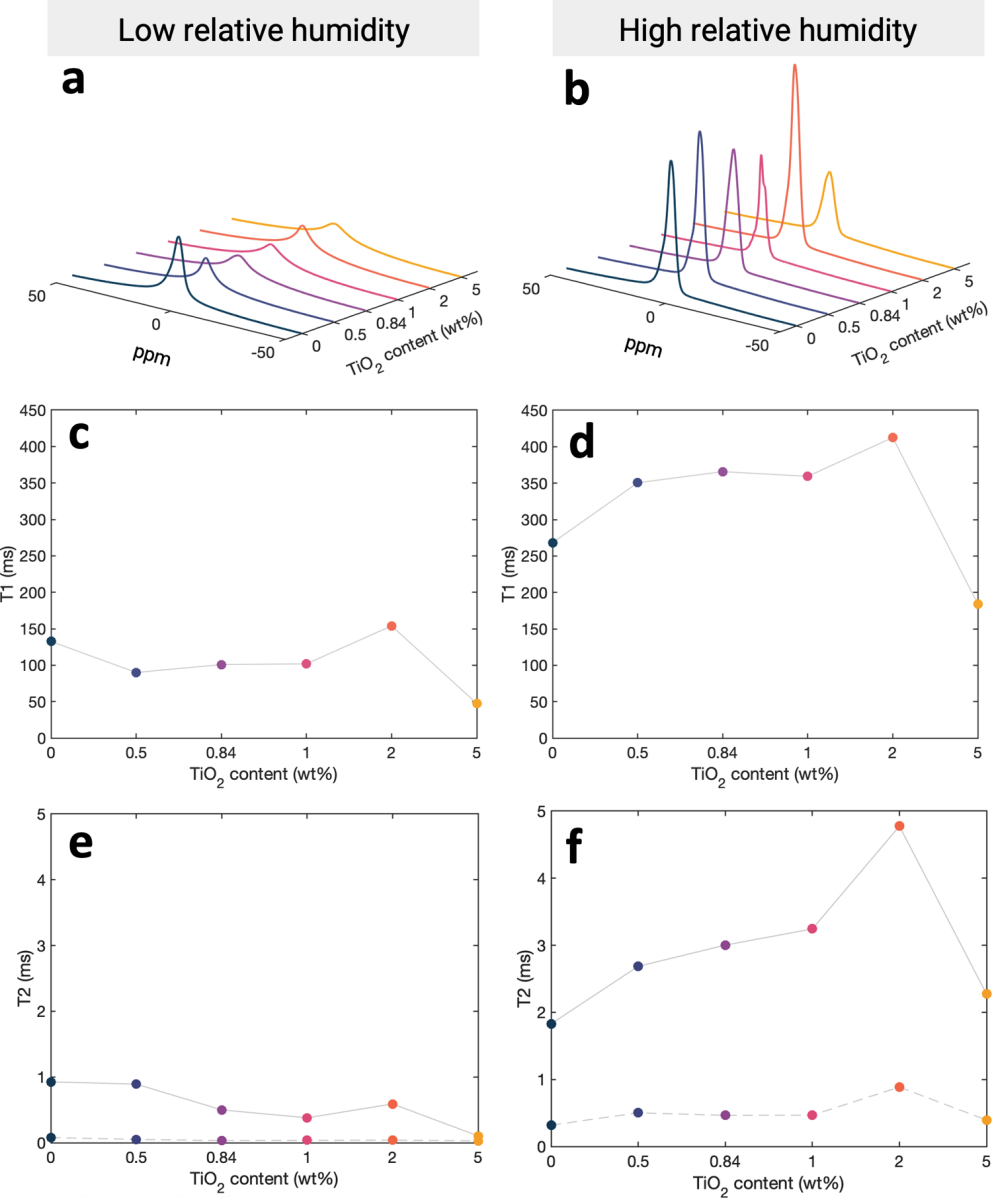
^1^H solid state NMR spectra comparing dry (a) and hydrated (b) Nafion samples for increasing TiO_2_ content; spectra are shown to scale. T_1_ versus TiO_2_ concentration comparing dry (c) versus hydrated (d) Nafion. T_2_ vs TiO_2_ concentration comparing dry (e) vs hydrated (f) Nafion. Solid lines correspond to component 1 and dashed lines to component 2.

To further investigate changing ionomer-water interactions, we measured molecular relaxation. We measure spin–lattice (T_1_) and spin–spin (T_2_) relaxation times under static conditions to understand how changes in the chemical environment with increasing TiO_2_ concentration affect water diffusion. The recovery of the longitudinal magnetic vector and the dephasing of the transverse magnetic vector are quantified by the T_1_ and T_2_ relaxation times. Here, these parameters are correlated with proton, and thereby water, mobility in the ionomer environment. Relaxation time constants are calculated from exponential fits to the signal data (eqs 8 and 9). To calculate T_1_, the signal data is well fit with a single component. However, to calculate T_2_, the data is not well fit with a single exponential, indicating the existence of more than one water population, and requiring a two-component model to accurately describe the molecular dynamics at short time scales. Fits of the transverse and longitudinal relaxation signals with eqs 8 and 9 with coefficients and adjusted R^2^ values are provided in Figures S12–S15.

T_1_ relaxation (also termed spin–lattice or longitudinal relaxation) probes the fast molecular motion on the MHz scale. T_1_ measurements provide information about the translational and rotational diffusion of molecules in the local chemical environment by quantifying the rate of energy transfer from the nuclear spin to neighboring molecules. We measure one component for the T_1_ proton relaxation in the Nafion–water system. Faster T_1_ times correspond to more strongly bound water that is adsorbed on the sulfonate group in the polymer matrix. The rate at which the system returns to thermal equilibrium is termed the relaxation rate and is the inverse of the relaxation time (*R*_1(2)_ = 1/*T*_1(2)_). The complexity of Nafion–water interactions is evidenced in the changing relaxation rates with increasing titania between low and high relative humidity environments (Figure 5c,d). Weakly bound water molecules take longer to relax, as they are shielded from the charged acid groups. The maximum relaxation time in the dry state is 153.9 ms at 2 wt % titania. At 2 wt % titania in the hydrated state, when water uptake is maximized, the proton T_1_ relaxation time reaches a maximum of 412.6 ms. This is a 54% increase from the T_1_ relaxation with 0 wt % titania.

T_2_ relaxation (also termed spin–spin or transverse relaxation) quantifies the rate of magnetization signal decay and is sensitive to molecular motions on shorter time scales of kHz. T_2_ relaxation times are a measure of water–ionomer exchange and are a function of hydration as the SO_3_H groups dissociate in the aqueous environment.^57^ Distances between water molecules and protons on the ionomer result in different mobilities and, thus, different T_2_ relaxation times. Water molecules that are far from the ionomer surface groups have very high mobility and therefore a slower relaxation rate, while water molecules at the surface of the ionomer are more restricted and have rapid relaxation rates. Multiexponential relaxation differentiates between bound water near the sulfonic acid groups and free water within the hydrophilic domains, as identified with PFG NMR. In the dry state, relaxation times decrease from 0.93 and 0.08 ms in pure Nafion as more TiO_2_ particles are added as the filler particles further hinder any proton movement (Figure 5e). With measurements of T_2_ in a high RH environment, the relaxation times for both identified components of water increase from 1.8 and 0.32 ms at 0 wt % titania up to 4.8 and 0.89 ms at 2 wt % titania, indicating that the presence of a TiO_2_ causes changes in the rates of water transport (Figure 5f). At 5 wt % titania, the T_2_ relaxation times are reduced to 2.3 and 0.39 ms.

## Conclusions

The addition of TiO_2_ to Nafion alters the water uptake and the diffusion of strongly and weakly bound water molecules. TiO_2_ particles enhance ionomer water uptake by up to 110% at a concentration of 2 wt %. Up to 2 wt % TiO_2_, water uptake is enhanced due to the hydrophilic nature of TiO_2_. However, at 5 wt % TiO_2_, hydration is reduced to an average water uptake of 12% as the filler particles occupy space in the hydrophilic transport channels. We identified two water diffusion coefficients in the Nafion–titania mixture through PFG NMR that correspond to two populations of water with different translational diffusion coefficients. The primary diffusion coefficient (*D*_1_) increases from 3.6 × 10^–10^ m^2^/s in Nafion to a maximum of 2.6 × 10^–9^ m^2^/s in Nafion+2 wt % TiO_2_. The secondary diffusion coefficient (*D*_2_) increases from 2.04 × 10^–11^ m^2^/s in Nafion to a maximum of 5.22 × 10^–11^ m^2^/s in Nafion+2 wt % TiO_2_. We observe that water molecules with diffusion coefficient *D*_2_ are more confined than those with diffusion coefficient *D*_1_ by measuring mean square displacement vs experimental diffusion time, as well as by differences in relaxation times (T_1_ and T_2_). Relaxation times highlight differences in water–ionomer interactions as water molecules diffuse and exchange with protons and resulting in faster relaxation times for more confined water molecules located near the ionomer. NMR relaxometry and diffusometry are valuable techniques for characterizing water diffusion in charged ionomers with the addition of inorganic fillers, as with these methods, we can distinguish between different components of water diffusion present in hydrated Nafion.

## Supplementary Material



## Notes

Tabulations of water uptake and water diffusion in hydrated Nafion with filler materials from the literature, DVS experimental timeline, SEM, adsorption–desorption isotherms, and coefficients and goodness of fit for T1 and T2 data (PDF)

## References

[1] Ling X., Bonn M., Domke K. F., Parekh S. H. (2019). Correlated interfacial
water transport and proton conductivity in perfluorosulfonic acid
membranes. Proc. Natl. Acad. Sci. U. S. A..

[2] Park H. B., Kamcev J., Robeson L. M., Elimelech M., Freeman B. D. (2017). Maximizing the right stuff: The trade-off
between membrane
permeability and selectivity. Science.

[3] Kamcev J., Galizia M., Benedetti F. M., Jang E. S., Paul D. R., Freeman B. D., Manning G. S. (2016). Partitioning
of mobile ions between
ion exchange polymers and aqueous salt solutions: importance of counter-ion
condensation. Physical chemistry chemical physics:
PCCP.

[4] Bye K. P., Galizia M. (2020). Fundamental origin of flux non-linearity in organic
solvent nanofiltration: Formulation of a thermodynamic/diffusion framework. J. Membr. Sci..

[5] Duan Q., Wang H., Benziger J. (2012). Transport of liquid
water through
Nafion membranes. J. Membr. Sci..

[6] Choe Y.-K., Tsuchida E., Ikeshoji T., Yamakawa S., Hyodo S.-A. (2008). Nature
of Water Transport and Electro-Osmosis in Nafion: Insights from First-Principles
Molecular Dynamics Simulations under an Electric Field. J. Phys. Chem. B.

[7] Amjadi M., Rowshanzamir S., Peighambardoust S., Hosseini M., Eikani M. (2010). Investigation
of physical properties and cell performance of Nafion/TiO2 nanocomposite
membranes for high temperature PEM fuel cells. Int. J. Hydrogen Energy.

[8] Ochi S., Kamishima O., Mizusaki J., Kawamura J. (2009). Investigation of proton
diffusion in Nafion®117 membrane by electrical conductivity and
NMR. Solid State Ionics.

[9] Ahmad S., Nawaz T., Ali A., Orhan M. F., Samreen A., Kannan A. M. (2022). An overview of proton
exchange membranes for fuel cells:
Materials and manufacturing. Int. J. Hydrogen
Energy.

[10] Prykhodko Y., Fatyeyeva K., Hespel L., Marais S. (2021). Progress in hybrid
composite Nafion®-based membranes for proton exchange fuel cell
application. Chemical Engineering Journal.

[11] Lin C.-C., Lien W.-F., Wang Y.-Z., Shiu H.-W., Lee C.-H. (2012). Preparation
and performance of sulfonated polyimide/Nafion multilayer membrane
for proton exchange membrane fuel cell. J. Power
Sources.

[12] Thampan T. M., Jalani N. H., Choi P., Datta R. (2005). Systematic Approach
to Design Higher Temperature Composite PEMs. J. Electrochem. Soc..

[13] Teixeira F.
C., de Sá A. I., Teixeira A. P., Rangel C. (2019). Nafion phosphonic acid
composite membranes for proton exchange membranes fuel cells. Appl. Surf. Sci..

[14] Zhao Q., Majsztrik P., Benziger J. (2011). Diffusion and Interfacial
Transport
of Water in Nafion. J. Phys. Chem. B.

[15] Bazrgar
Bajestani M., Mousavi S. A. (2016). Effect of casting solvent on the
characteristics of Nafion/TiO2 nanocomposite membranes for microbial
fuel cell application. Int. J. Hydrogen Energy.

[16] Jian-hua T., Peng-fei G., Zhi-yuan Z., Wen-hui L., Zhong-qiang S. (2008). Preparation
and performance evaluation of a Nafion-TiO2 composite membrane for
PEMFCs. Int. J. Hydrogen Energy.

[17] Jalani N. H., Dunn K., Datta R. (2005). Synthesis
and characterization of
Nafion®-MO2 (M = Zr, Si, Ti) nanocomposite membranes for higher
temperature PEM fuel cells. Electrochim. Acta.

[18] Rodgers M. P., Shi Z., Holdcroft S. (2008). Transport
properties of composite membranes containing
silicon dioxide and Nafion®. J. Membr.
Sci..

[19] Guzmán C., Alvarez A., Herrera O., Nava R., Ledesma-Garcia J., Godínez L. A., Arriaga L., Mérida W. (2011). Water Transport
in Composite Membranes Containing Silica: Temperature and Relative
Humidity Effects. Int. J. Electrochem. Sci..

[20] Ye G., Hayden C. A., Goward G. R. (2007). Proton
Dynamics of Nafion and Nafion/SiO2
Composites by Solid State NMR and Pulse Field Gradient NMR. Macromolecules.

[21] Kreuer K.-D., Dippel T., Meyer W., Maier J. (1992). Nafion Membranes: Molecular
Diffusion, Proton Conductivity and Proton Conduction Mechanism. MRS Online Proc. Libr..

[22] Perrin J.-C., Lyonnard S., Volino F. (2007). Quasielastic
Neutron Scattering Study
of Water Dynamics in Hydrated Nafion Membranes. J. Phys. Chem. C.

[23] Burnett D. J., Garcia A. R., Thielmann F. (2006). Measuring moisture sorption and diffusion
kinetics on proton exchange membranes using a gravimetric vapor sorption
apparatus. J. Power Sources.

[24] Majsztrik P. W., Satterfield M. B., Bocarsly A. B., Benziger J. B. (2007). Water sorption,
desorption and transport in Nafion membranes. J. Membr. Sci..

[25] Wakai C., Shimoaka T., Hasegawa T. (2015). 1H NMR Analysis of
Water Freezing
in Nanospace Involved in a Nafion Membrane. J. Phys. Chem. B.

[26] D’Agostino C., Mitchell J., Mantle M. D., Gladden L. F. (2014). Interpretation
of
NMR Relaxation as a Tool for Characterising the Adsorption Strength
of Liquids inside Porous Materials. *Chemistry–A*. European Journal.

[27] Hosseinpour S., Tang F., Wang F., Livingstone R. A., Schlegel S. J., Ohto T., Bonn M., Nagata Y., Backus E. H. G. (2017). Chemisorbed and Physisorbed Water
at the TiO2/Water
Interface. J. Phys. Chem. Lett..

[28] Karimi M. B., Mohammadi F., Hooshyari K. (2019). Recent approaches to improve Nafion
performance for fuel cell applications: A review. Int. J. Hydrogen Energy.

[29] Agmon N. (1995). The Grotthuss
mechanism. Chem. Phys. Lett..

[30] Zhu L.-Y., Li Y.-C., Liu J., He J., Wang L.-Y., Lei J.-D. (2022). Recent developments in high-performance
Nafion membranes
for hydrogen fuel cells applications. Petroleum
Science.

[31] Hammer R., Schönhoff M., Hansen M. R. (2019). Comprehensive picture of water dynamics
in nafion membranes at different levels of hydration. J. Phys. Chem. B.

[32] Privalov A. F., Sinitsyn V. V., Vogel M. (2023). Transport mechanism
in Nafion revealed
by detailed comparison of 1H and 17O nuclear magnetic resonance diffusion
coefficients. J. Phys. Chem. Lett..

[33] Gebel G. (2000). Structural
evolution of water swollen perfluorosulfonated ionomers from dry membrane
to solution. Polymer.

[34] Mauritz K. A., Moore R. B. (2004). State of Understanding
of Nafion. Chem. Rev..

[35] Robert M., Kaddouri A. E., Perrin J.-C., Leclerc S., Lottin O. (2018). Towards a
NMR-Based Method for Characterizing the Degradation of Nafion XL Membranes
for PEMFC. J. Electrochem. Soc..

[36] Nicotera I., Coppola L., Rossi C. O., Youssry M., Ranieri G. A. (2009). NMR Investigation
of the Dynamics of Confined Water in Nafion-Based Electrolyte Membranes
at Subfreezing Temperatures. J. Phys. Chem.
B.

[37] Tasaka M., Suzuki S., Ogawa Y., Kamaya M. (1988). Freezing and nonfreezing
water in charged membranes. J. Membr. Sci..

[38] Ludlam G. A. H., Gnaniah S. J. P., Degl’Innocenti R., Gupta G., Wain A. J., Lin H. (2024). Measurement of Water
Uptake and States in Nafion Membranes Using Humidity-Controlled Terahertz
Time-Domain Spectroscopy. ACS Sustainable Chem.
Eng..

[39] Zimm B. H., Lundberg J. L. (1956). Sorption of vapors by high polymers. J. Phys. Chem..

[40] Li Y., Nguyen Q. T., Buquet C. L., Langevin D., Legras M., Marais S. (2013). Water sorption in Nafion®membranes analyzed with
an improved dual-mode sorption model—Structure/property relationships. J. Membr. Sci..

[41] Brunauer S., Emmett P. H., Teller E. (1938). Adsorption of Gases
in Multimolecular
Layers. J. Am. Chem. Soc..

[42] Guggenheim, E. Application; Clarendon Press: Oxford, 1966.

[43] Feng H. (2007). Modeling of
vapor sorption in glassy polymers using a new dual mode sorption model
based on multilayer sorption theory. Polymer.

[44] Young J. F. (1967). Humidity
control in the laboratory using salt solutions—a review. Journal of Applied Chemistry.

[45] Stejskal E. O., Tanner J. E. (1965). Spin diffusion measurements:
spin echoes in the presence
of a time-dependent field gradient. J. Chem.
Phys..

[46] Price, W. S. NMR studies of translational motion: Principles and applications, 1st ed.; Cambridge University Press, 2009.

[47] Abbaszadeh M., Garell M., Choi J. I., Chen Y., Leisen J., Jang S. S., Hu Y.-Y., Hatzell M. C. (2023). Unraveling Water
and Salt Transport in Polyamide with Nuclear Magnetic Resonance Spectroscopy. ACS Materials Letters.

[48] Kusoglu, A. ; Weber, A. Z. Polymers for Energy Storage and Delivery: Polyelectrolytes for Batteries and Fuel Cells, 2012; Chapter 11, pp 175–199.

[49] Reif B., Ashbrook S. E., Emsley L., Hong M. (2021). Solid-state NMR spectroscopy. Nat. Rev. Methods
Primers.

[50] Ma Z., Jiang R., Myers M. E., Thompson E. L., Gittleman C. S. (2011). NMR studies
of proton transport in fuel cell membranes at sub-freezing conditions. J. Mater. Chem..

[51] Yoo H., Paranji R., Pollack G. H. (2011). Impact of Hydrophilic Surfaces on
Interfacial Water Dynamics Probed with NMR Spectroscopy. J. Phys. Chem. Lett..

[52] Engelke S., Marbella L. E., Trease N. M., Volder M. D., Grey C. P. (2019). Three-dimensional
pulsed field gradient NMR measurements of self-diffusion in anisotropic
materials for energy storage applications. Phys.
Chem. Chem. Phys..

[53] Andrada H. E., Franzoni M. B., Carreras A. C., Chávez F. V. (2018). Dynamics
and spatial distribution of water in Nafion 117 membrane investigated
by NMR spin-spin relaxation. Int. J. Hydrogen
Energy.

[54] Tsimpanogiannis I. N., Moultos O. A., Franco L. F. M., de M Spera M. B., Erdös M., Economou I. G. (2019). Self-diffusion coefficient of bulk
and confined water: a critical review of classical molecular simulation
studies. Mol. Simul..

[55] Oener S. Z., Foster M. J., Boettcher S. W. (2020). Accelerating
water dissociation in
bipolar membranes and for electrocatalysis. Science.

[56] Shimoaka T., Wakai C., Sakabe T., Yamazaki S., Hasegawa T. (2015). Hydration
structure of strongly bound water on the sulfonic acid group in a
Nafion membrane studied by infrared spectroscopy and quantum chemical
calculation. Phys. Chem. Chem. Phys..

[57] MacMillan B., Sharp A. R., Armstrong R. L. (1999). An n.m.r.
investigation of the dynamical
characteristics of water absorbed in Nafion. Polymer.

