# Supplementary material for: Impact of Titanium Dioxide on Water Uptake and Diffusion in Nafion
Source: J Phys Chem C Nanomater Interfaces. 2025 May 19;129(21):9867–76. doi: 10.1021/acs.jpcc.5c00822 (PMC12128095; doi:10.1021/acs.jpcc.5c00822)
Supplement: Supplementary file 1 [file jp5c00822_si_001.pdf]

Supporting Information: Impact of Titanium Dioxide on Water Uptake and Diffusion in Nafion

Madeline Garell,[†] Natechanok Yutthasaksunthorn,[‡] Hakhyeon Song,[†] Johannes Leisen,[¶] and Marta C. Hatzell[*,†,§]

†*George W. Woodruff School of Mechanical Engineering*

‡*School of Chemical and Biomolecular Engineering*

¶*School of Chemistry and Biochemistry*

§*School of Chemical and Biomolecular Engineering, Georgia Institute of Technology, Atlanta, GA, 30332 USA*

E-mail: marta.hatzell@me.gatech.edu

Table S1: Water uptake measurements from literature in hydrated Nafion measured at 25 C

Ionomer	Inorganic Filler	Water Uptake	Reference
Nafion 117		31%	[1]
Nafion 117	3 wt % TiO_2	51%	[1]
Nafion 117	5 wt % TiO_2	40%	[1]
Nafion 117	10 wt % TiO_2	28%	[1]
Nafion 112		30%	[2]
Nafion 112	1 wt % TiO_2	51%	[2]
Nafion		19.8%	[3]
Nafion	1 wt % SiO_2	24.2%	[3]
Nafion	3 wt % SiO_2	30.2%	[3]
Nafion		21%	[4]
Nafion	1 wt% HfO_2	44%	[4]
Nafion 115		32%	[5]
Nafion	10 wt % TiO_2	34%	[5]
Nafion	10 wt % SiO_2	34%	[5]
Nafion	10 wt % WO_3	37%	[5]
Nafion	14.3 wt % SiO_2-PWA	38%	[5]
Nafion		37%	[6]
Nafion	2.5 wt % TiO_2	39%	[6]
Nafion	2.5 wt % SiO_2	47%	[6]
Nafion 212		23.7%	[7]
Nafion	1 wt % ZrNT	28.9%	[7]
Nafion	1.5 wt % ZrNT	31.9%	[7]
Nafion	2 wt % ZrNT	26.7%	[7]
Nafion resin		19%	[8]
Nafion resin	3 wt % CS-SiO_2	24%	[8]
Nafion resin	6 wt % CS-SiO_2	28%	[8]
Nafion resin	9 wt % CS-SiO_2	32%	[8]
Nafion resin	12 wt % CS-SiO_2	35%	[8]
Nafion 115		35%	[9]
Nafion 115	11.1 wt % TiO_2	31%	[9]
Nafion 115	16.7 wt % TiO_2	26%	[9]
Nafion 115	21.6 wt % TiO_2	27%	[9]
Nafion 115	26.4 wt % TiO_2	23%	[9]
Nafion 117		31.5%	[10]
Nafion (commercial)		27%	[11]
Nafion (recast)		20%	[11]
Nafion (recast)	3 wt % TiO_2	29%	[11]

Table S2: Water diffusion measurements from literature in hydrated Nafion measured at 25 C

Measurement technique	Ionomer	Filler	D_1 (m^2/s)	D_2 (m^2/s)	Reference
Water permeation	Nafion 1110		3×10^{-10}		12
Water permeation	Nafion		2.2×10^{-10}		13
Adsorption isotherms	Nafion 1110		3×10^{-9}		12
Adsorption isotherms	Nafion 117		1×10^{-10}		14
Water sorption from vapor	Nafion		2×10^{-12}		15
Water sorption from liquid	Nafion		1.8×10^{-10}		15
Desorption	Nafion		1.1×10^{-11}		15
Electrical conductivity	Nafion 117		10^{-10} - 10^{-11}		16
NMR	Nafion 117		10^{-10} - 10^{-11}		16
NMR	Nafion		1 - 19×10^{-10}		17
NMR	Nafion		7.7×10^{-10}	9.4×10^{-11}	18
NMR	Nafion		4.83×10^{-10}		19
NMR	Nafion	1-2.8 wt % SiO_2	0.96 - 2.15×10^{-11}		19
NMR	Nafion 117		5.2×10^{-10}		20
NMR	Nafion		4×10^{-10}		21
NMR	Nafion	5 wt % $SZrO_2$	6×10^{-10}		21
NMR	Nafion		5×10^{-10}		21
NMR	Nafion	5 wt % ZrO_2	8×10^{-10}		21
NMR	Nafion 212		2.003×10^{-9}		7
NMR	Nafion 212	1.5 wt % ZrNT	3.272×10^{-9}		7
NMR	Nafion		1.8×10^{-10}		22
NMR	Nafion		2.5×10^{-10}		17
tATR-FTIR Spectroscopy	Nafion (unannealed)		8.3×10^{-10}		23
tATR-FTIR Spectroscopy	Nafion (annealed)		22.4×10^{-10}		23
tATR-FTIR Spectroscopy	Nafion (unannealed)	4 wt % SiO_2	16.6×10^{-10}		23
tATR-FTIR Spectroscopy	Nafion (annealed)	4 wt % SiO_2	5.7×10^{-10}		23
tATR-FTIR Spectroscopy	Nafion	10 wt % SiO_2	19.3×10^{-10}		23
QENS	Nafion 117		2.14×10^{-9}	1.04×10^{-9}	24
QENS	Nafion 112		2×10^{-9}	0.5×10^{-9}	25

Two distinct water diffusion coefficients have been measured in Nafion using quasi-elastic neutron scattering (QENS)[26] and nuclear magnetic resonance (NMR).[18,20] Additional tabulations of water diffusion at different conditions (with varying temperature, water activity, relative humidity) in Nafion as measured by a variety of techniques (liquid water sorption, water vapor sorption, adsorption-desorption experiments, permeation, NMR, QENS, radiotracer, and SANS) have been compiled by Kusoglu and Weber, 2012,[27] Majsztrik et al., 2007,[12] and Zhao et al., 2011.[28]

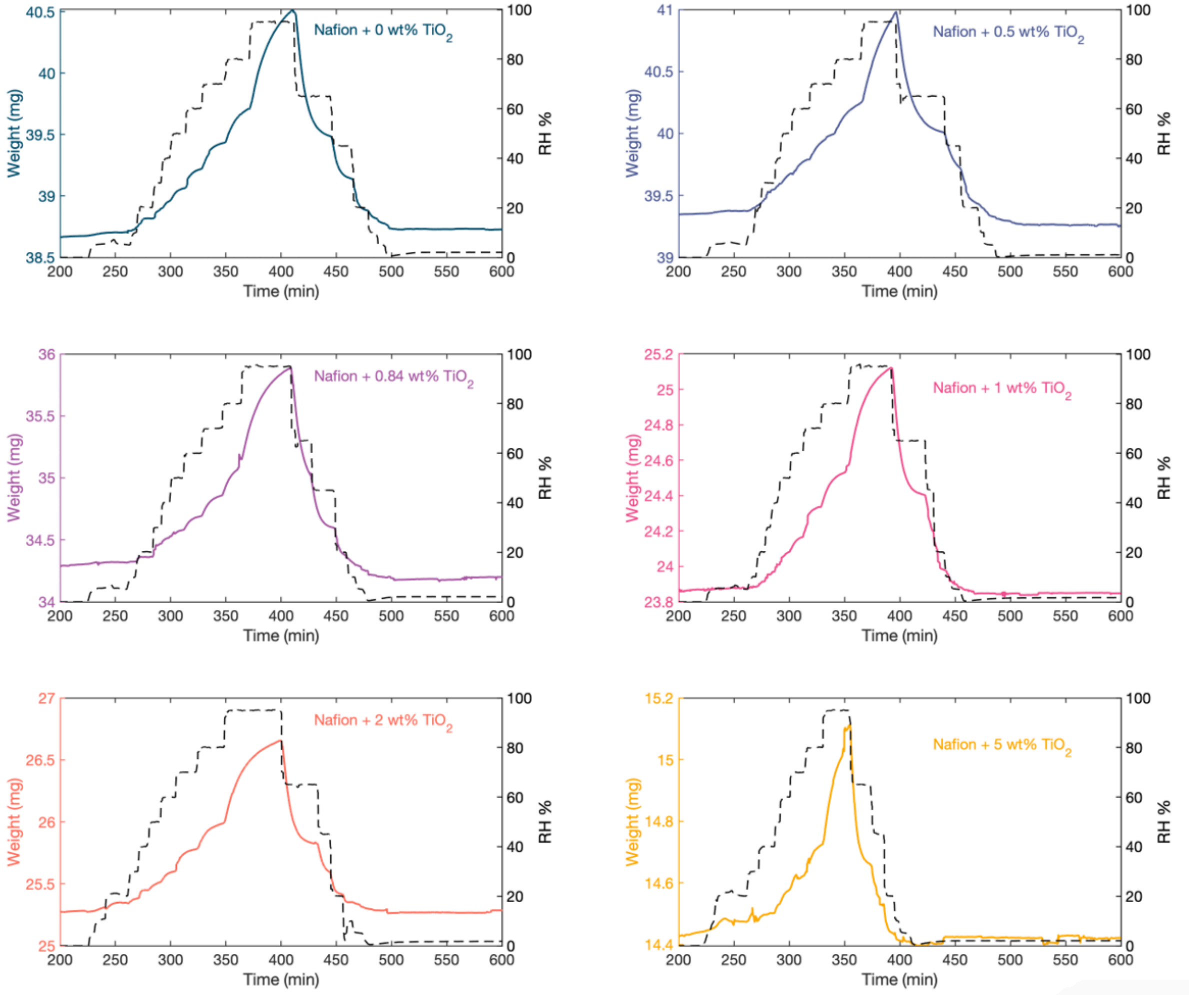

Figure S1: Dynamic vapor sorption experiment timeline

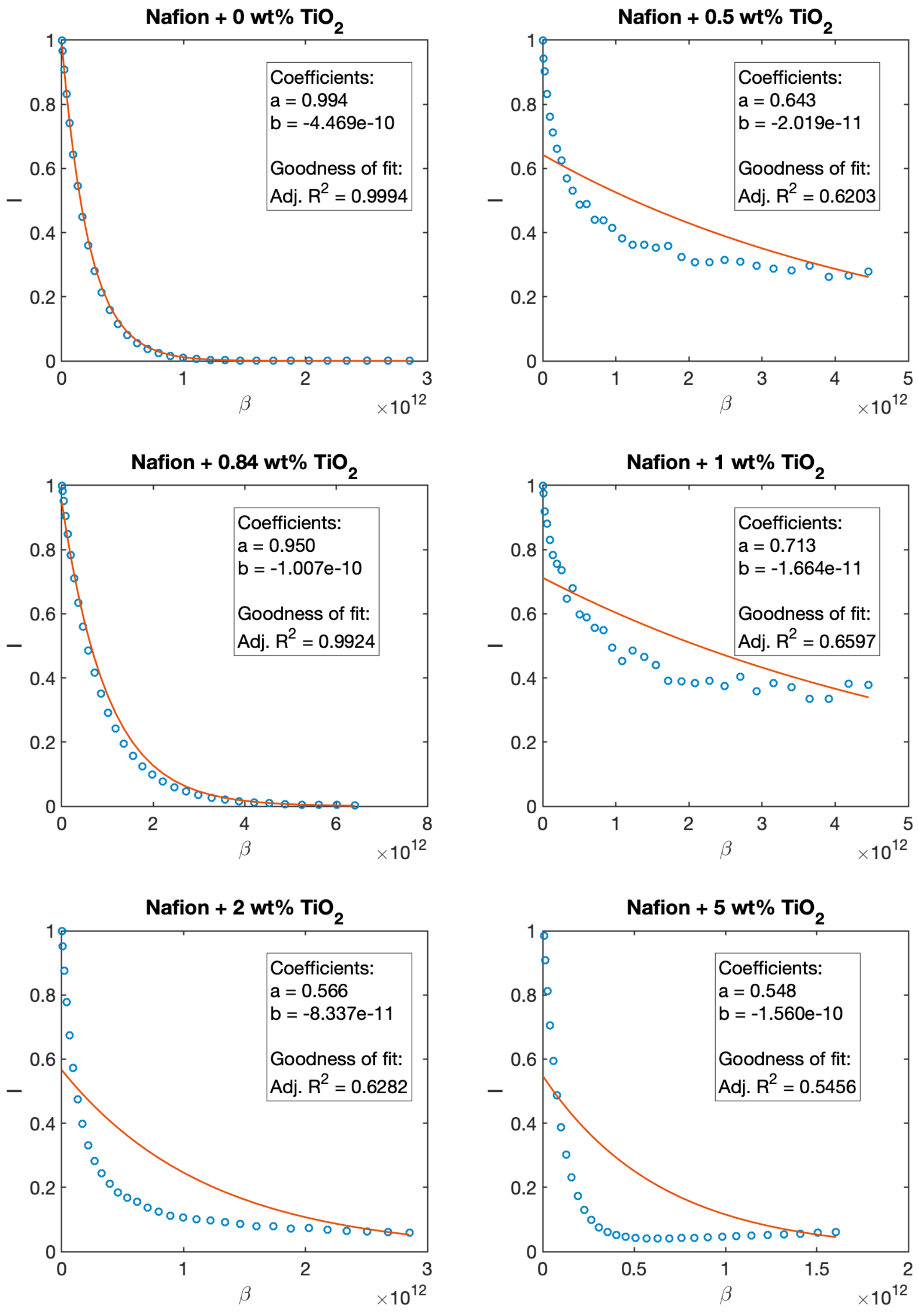

Figure S2: Single exponential fit of PFG NMR signal attenuation.

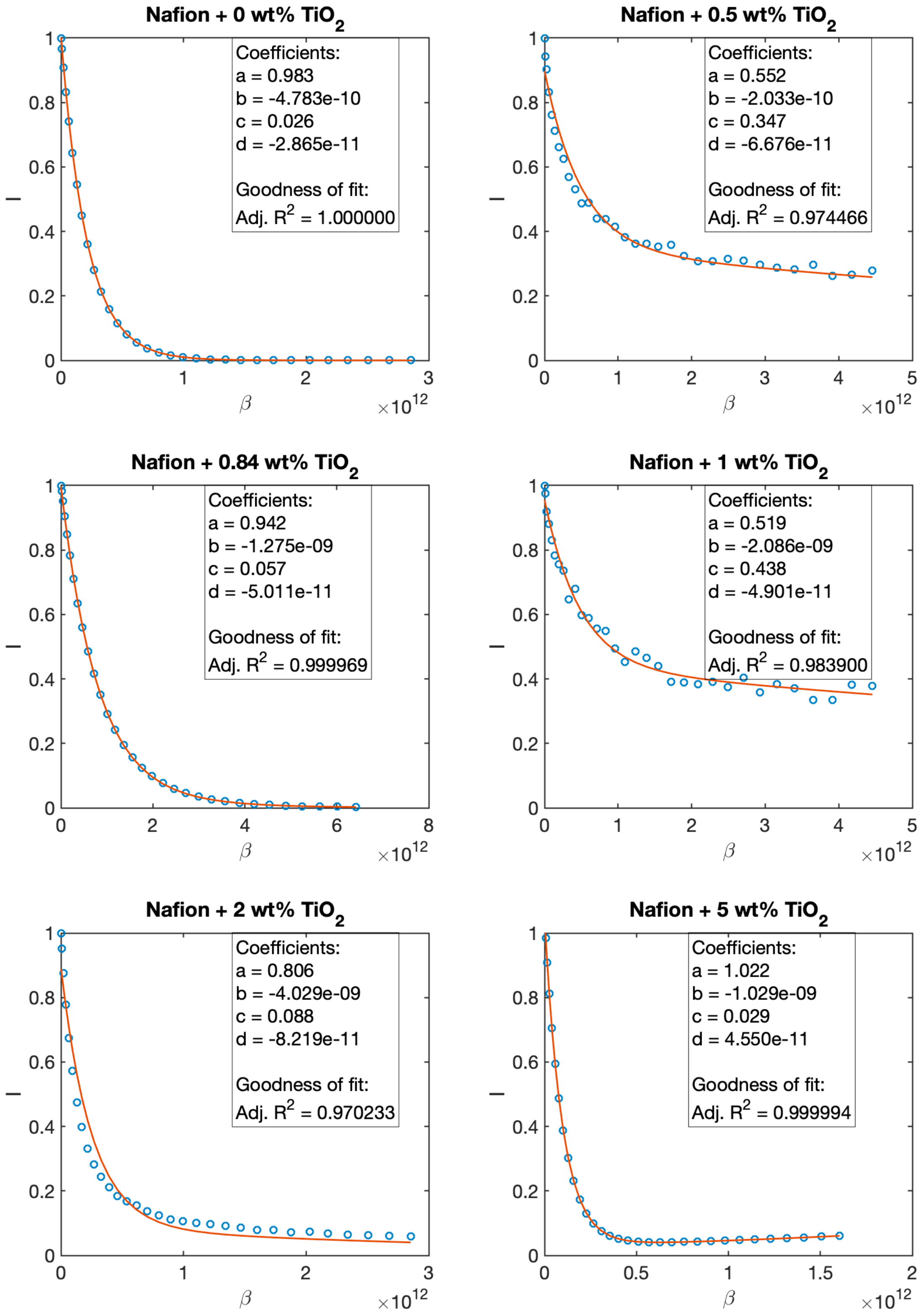

Figure S3: Double exponential fit of PFG NMR signal attenuation.

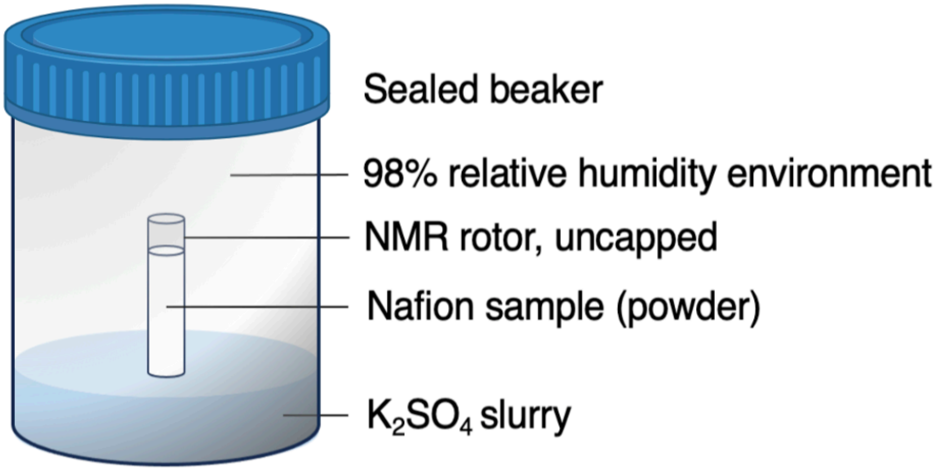

Figure S4: Experimental setup to equilibrate solid state NMR samples with 98% relative humidity environment

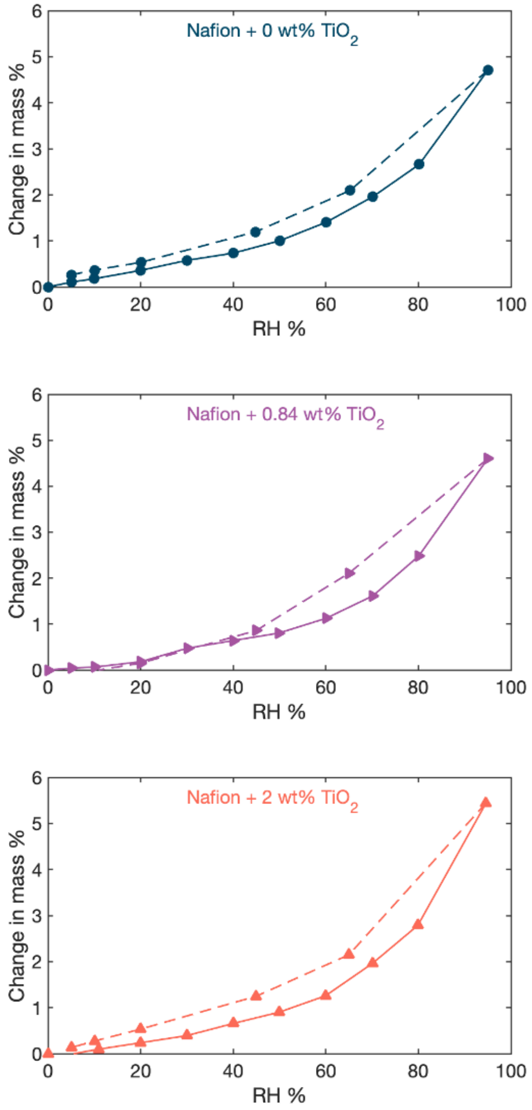

Figure S5: Adsorption-desorption isotherms

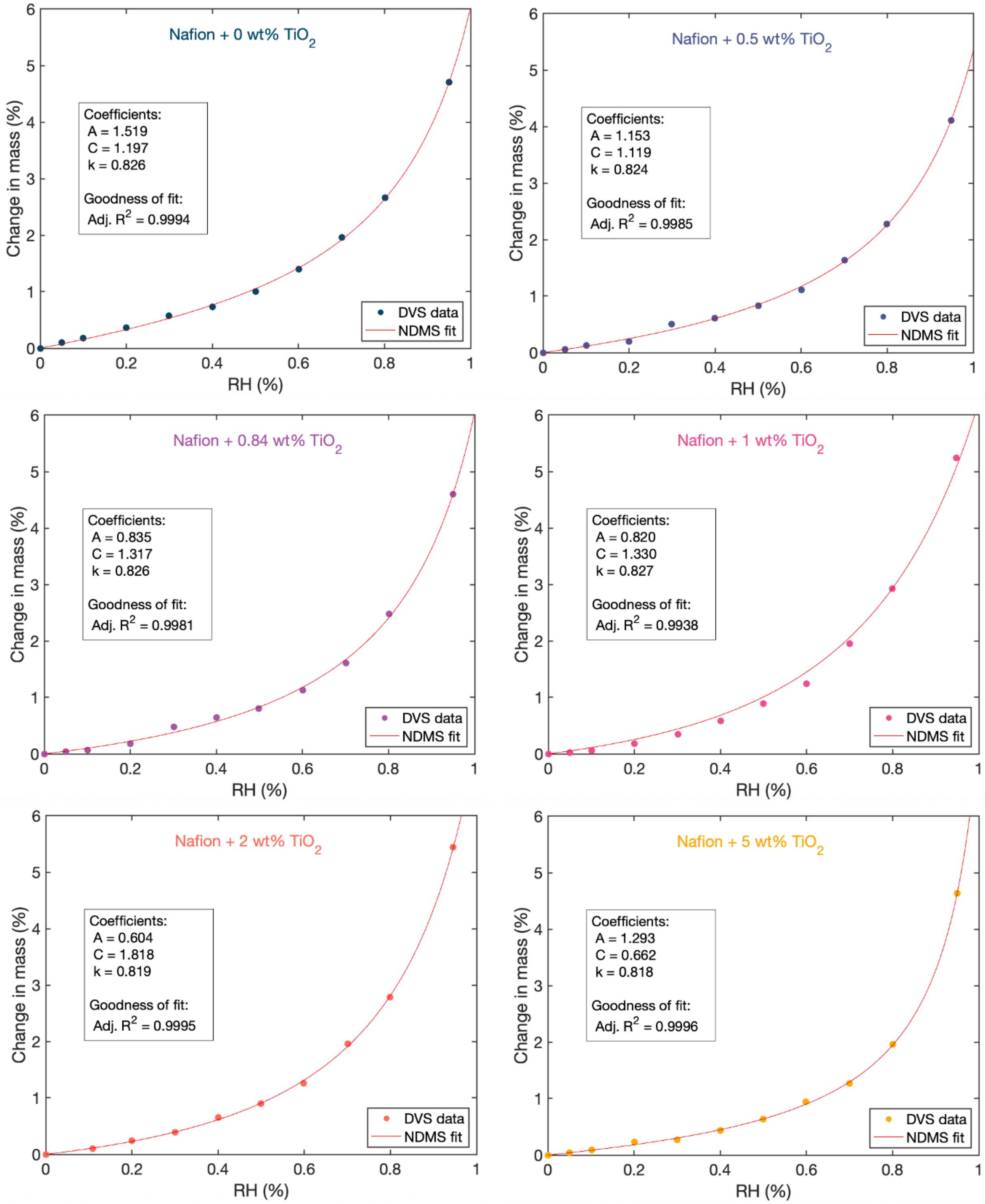

Figure S6: Dynamic vapor sorption (DVS) data and Dual Mode Sorption (DMS) model fit for each Nafion + TiO_2 sample.

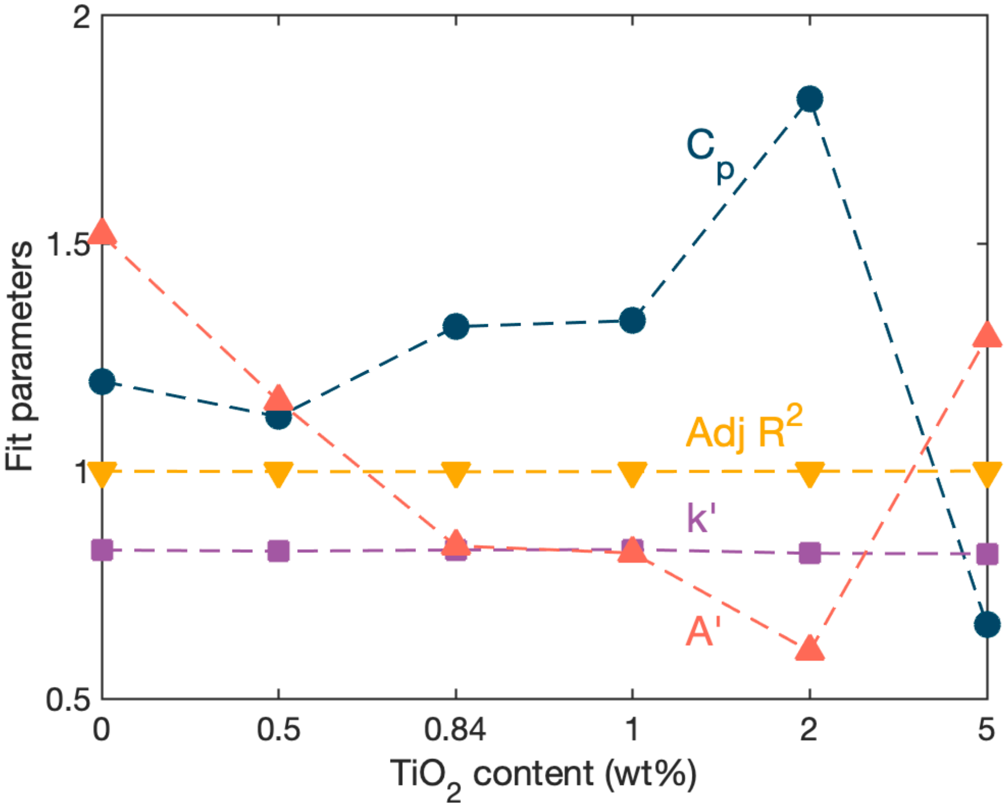

Figure S7: Calculated parameters to fit adsorption isotherms using the dual mode sorption model

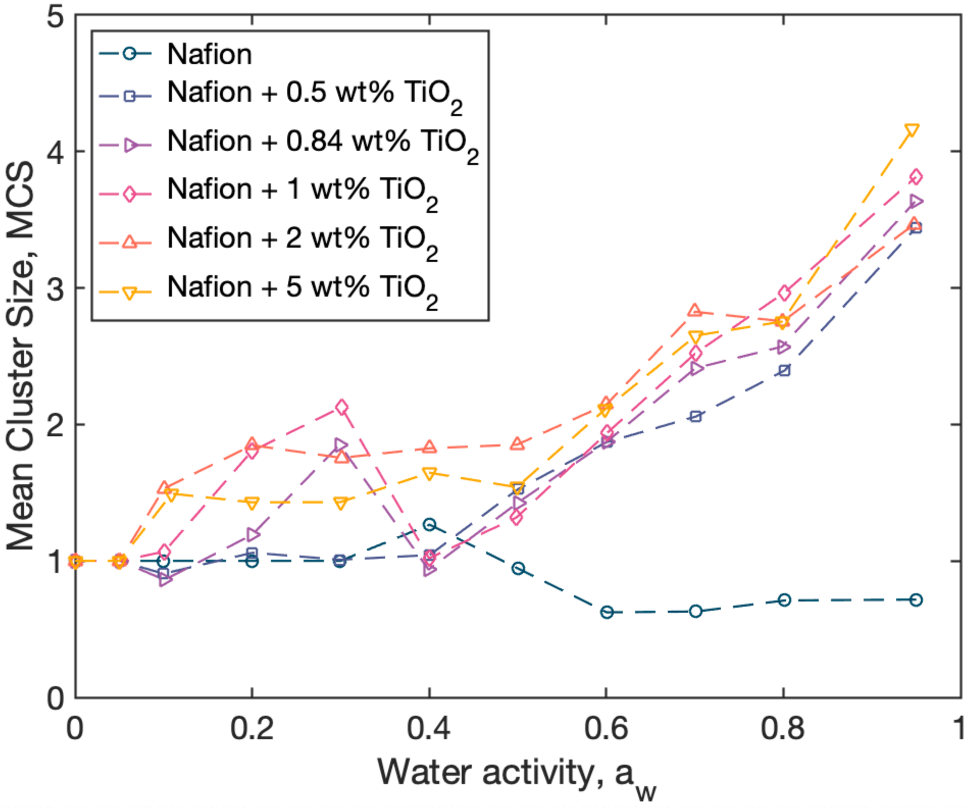

Figure S8: Mean cluster size (MCS) as evaluated from the Zimm-Lundberg function and dynamic vapor sorption data.

SEM thickness measurement

Thickness is measured from samples of the same membranes used for dynamic vapor sorption, all of which were hydrated to 50% relative humidity. Correlation between TiO_2 concentration and sample thickness is not required as the thickness is included in the calculation of the water diffusion coefficient.

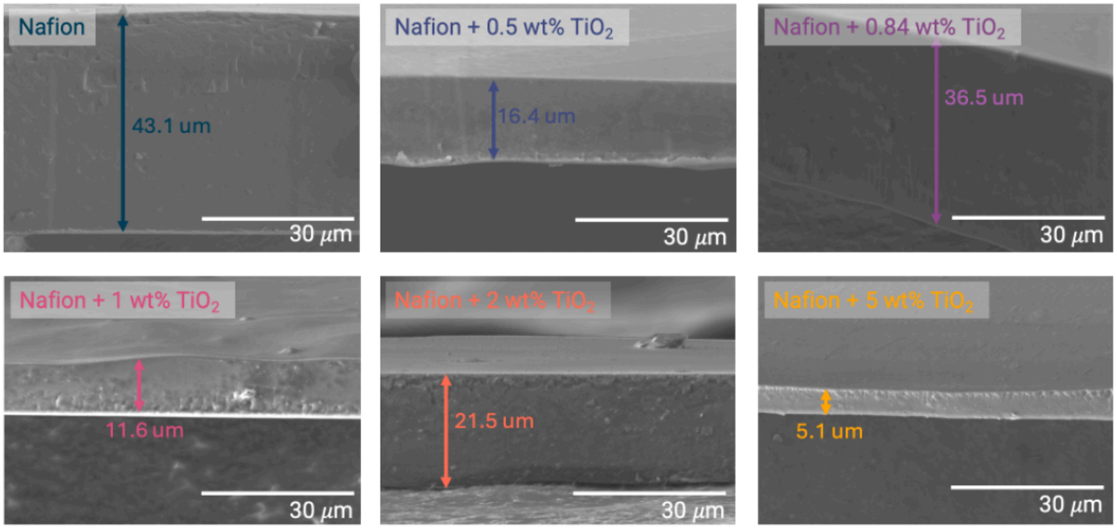

Figure S9: Cross section SEM of solution cast Nafion-TiO_2 membranes

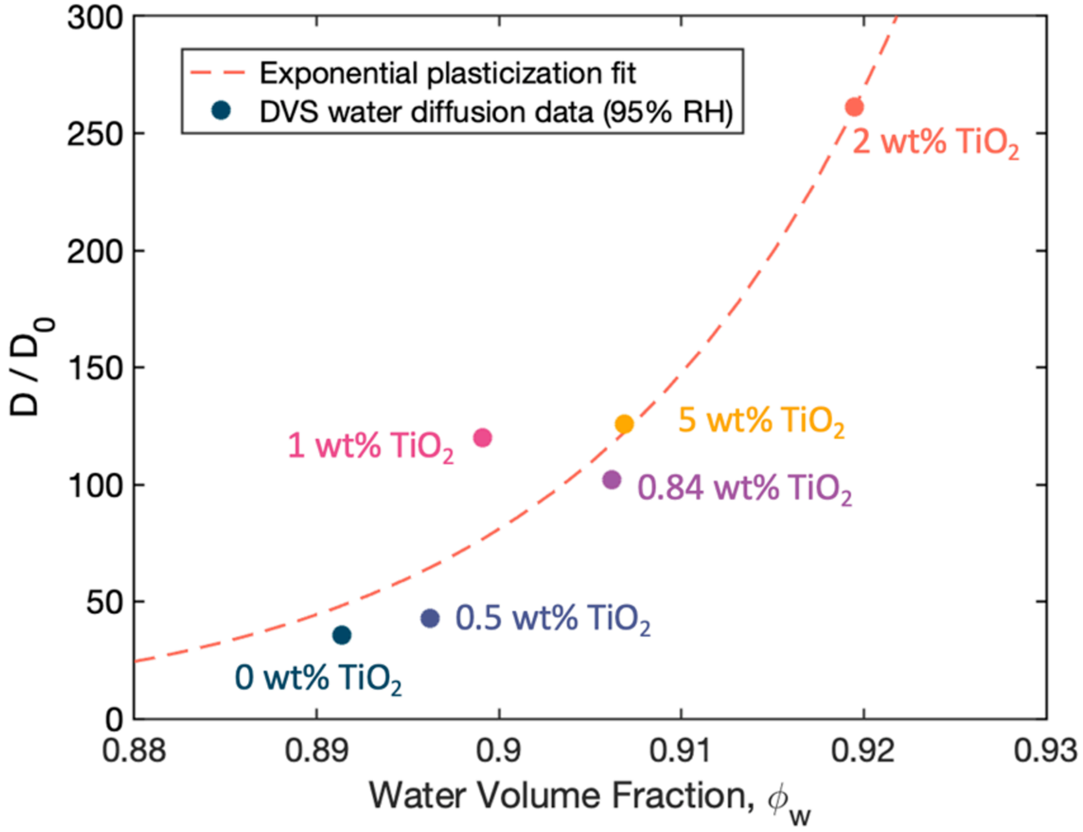

Figure S10: Exponential relationship between diffusivity and water volume fraction in the hydrated (95% RH) ionomer. D is the diffusion of water in the hydrated ionomer and D_0 is the diffusion of water in the dry ionomer.

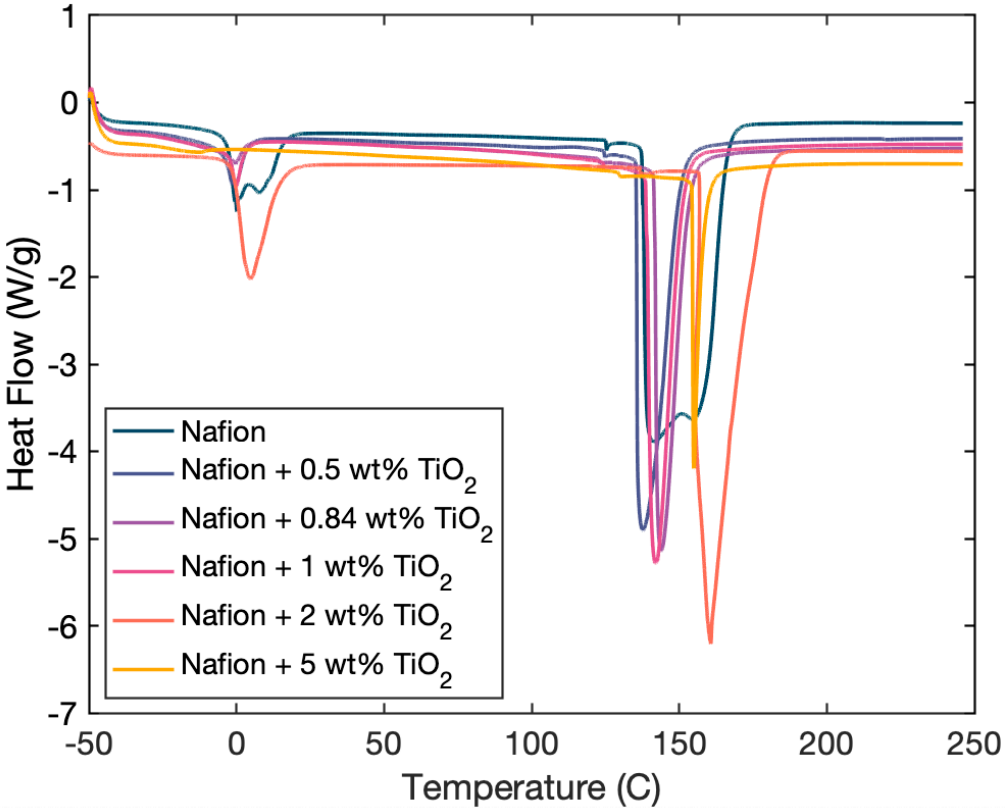

Figure S11: DSC thermogram for fully hydrated Naifon-TiO_2 samples.

T_1 and T_2 relaxation

T_1 relaxation times are calculated from a one component fit (Eq. 1a) while T_2 relaxation times are calculated from a two component fit (Eq. 1b).

$$I_{T_1} = I\left(1 - 2A\exp\left(\frac{-t}{T_1}\right)\right) \tag{1a}$$

$$I_{T_2} = a\exp\left(bt\right) + c\exp\left(dt\right) \tag{1b}$$

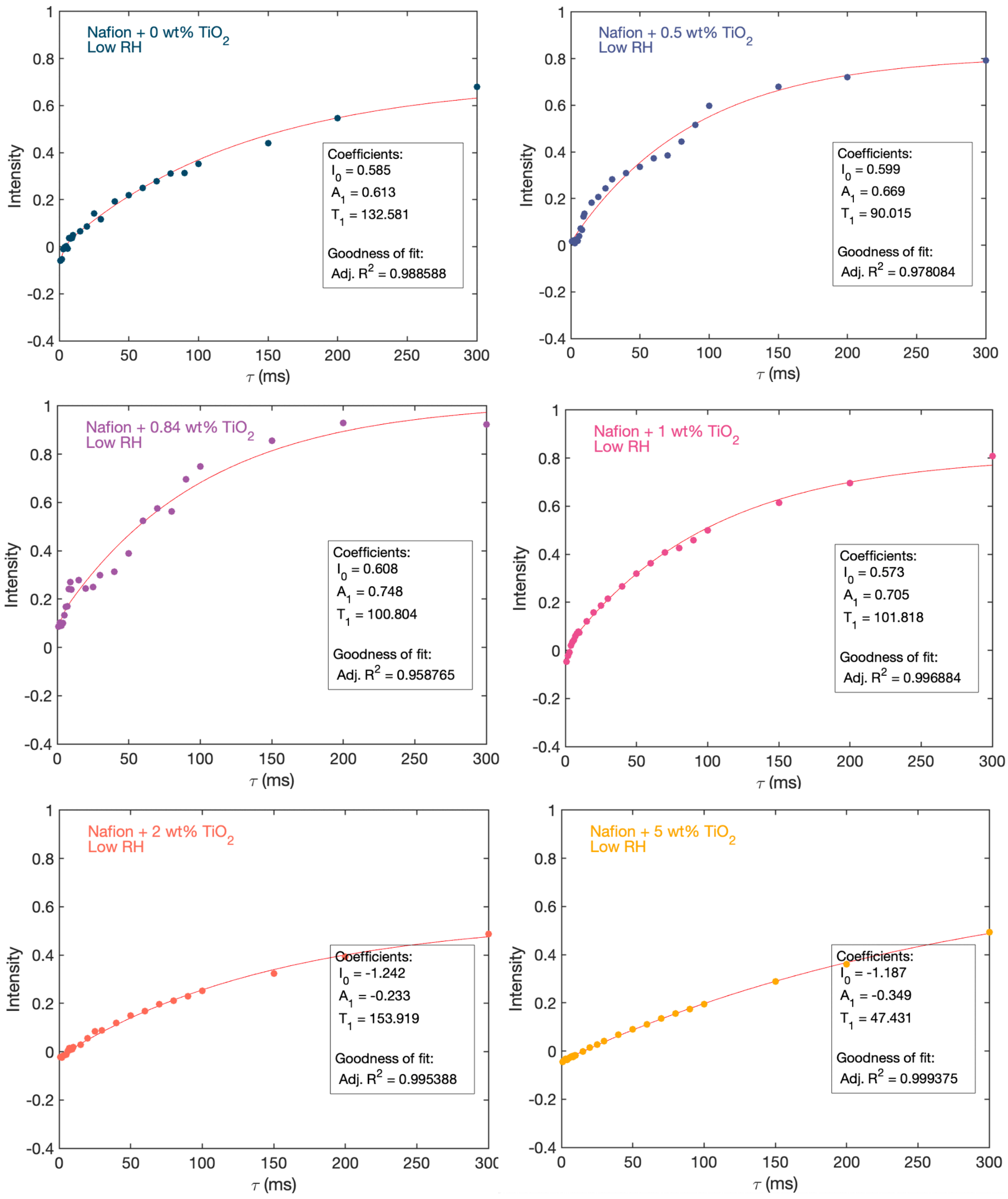

Figure S12: ^{1}H NMR T_1 inversion recovery fits for low relative humidity samples

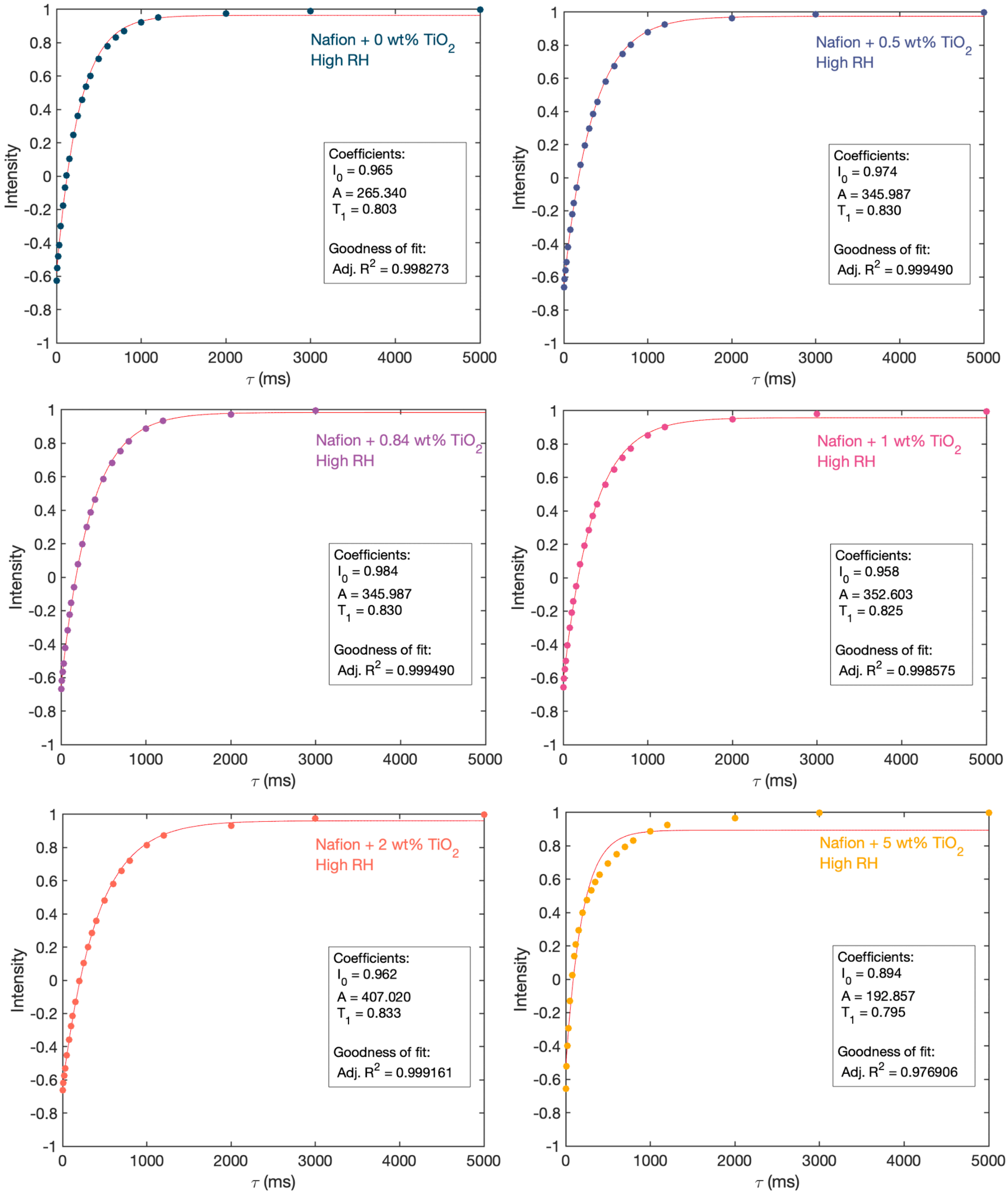

Figure S13: ^{1}H NMR T_1 inversion recovery fits for high relative humidity samples

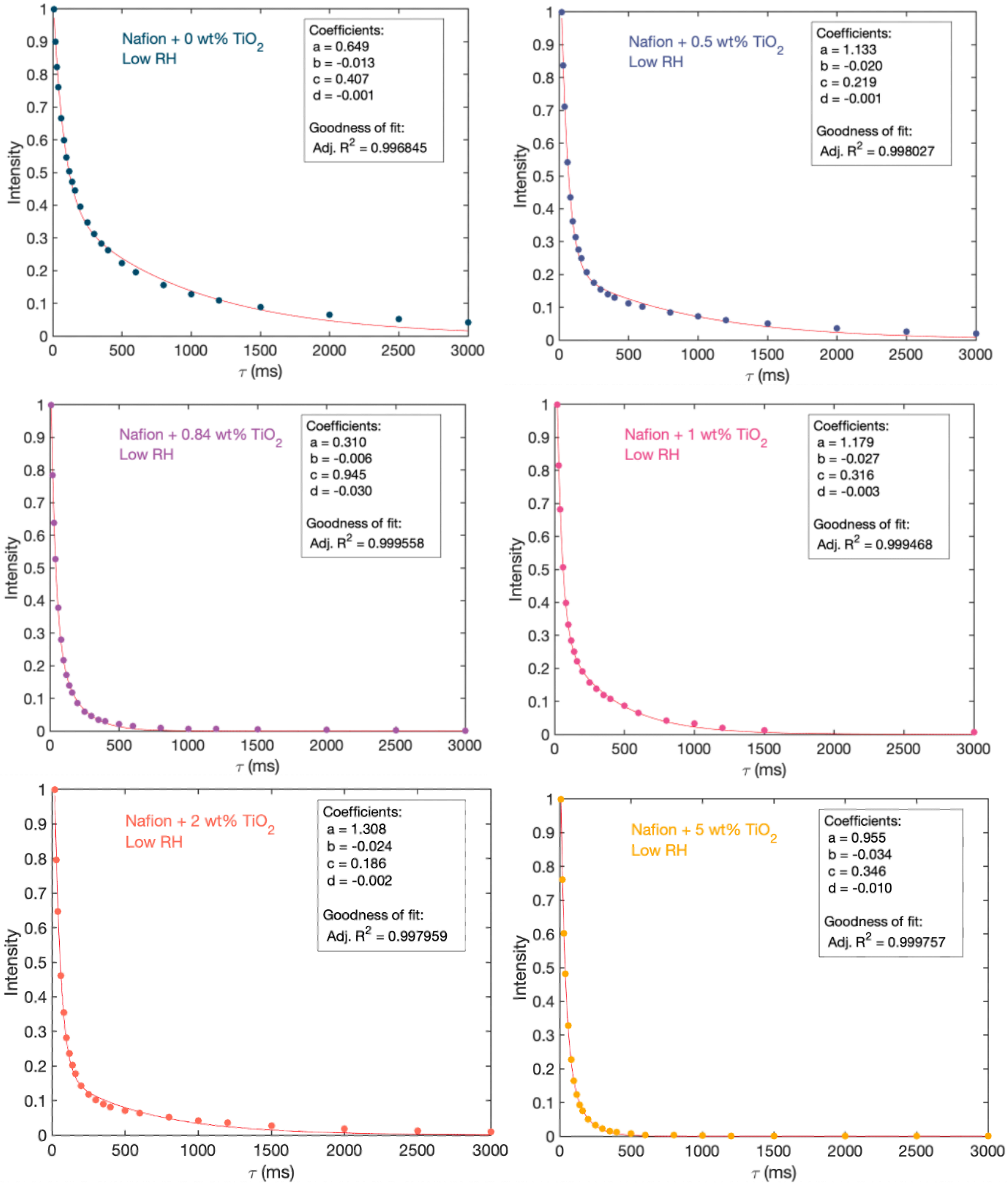

Figure S14: ^{1}H NMR T_2 decay fits for low relative humidity samples

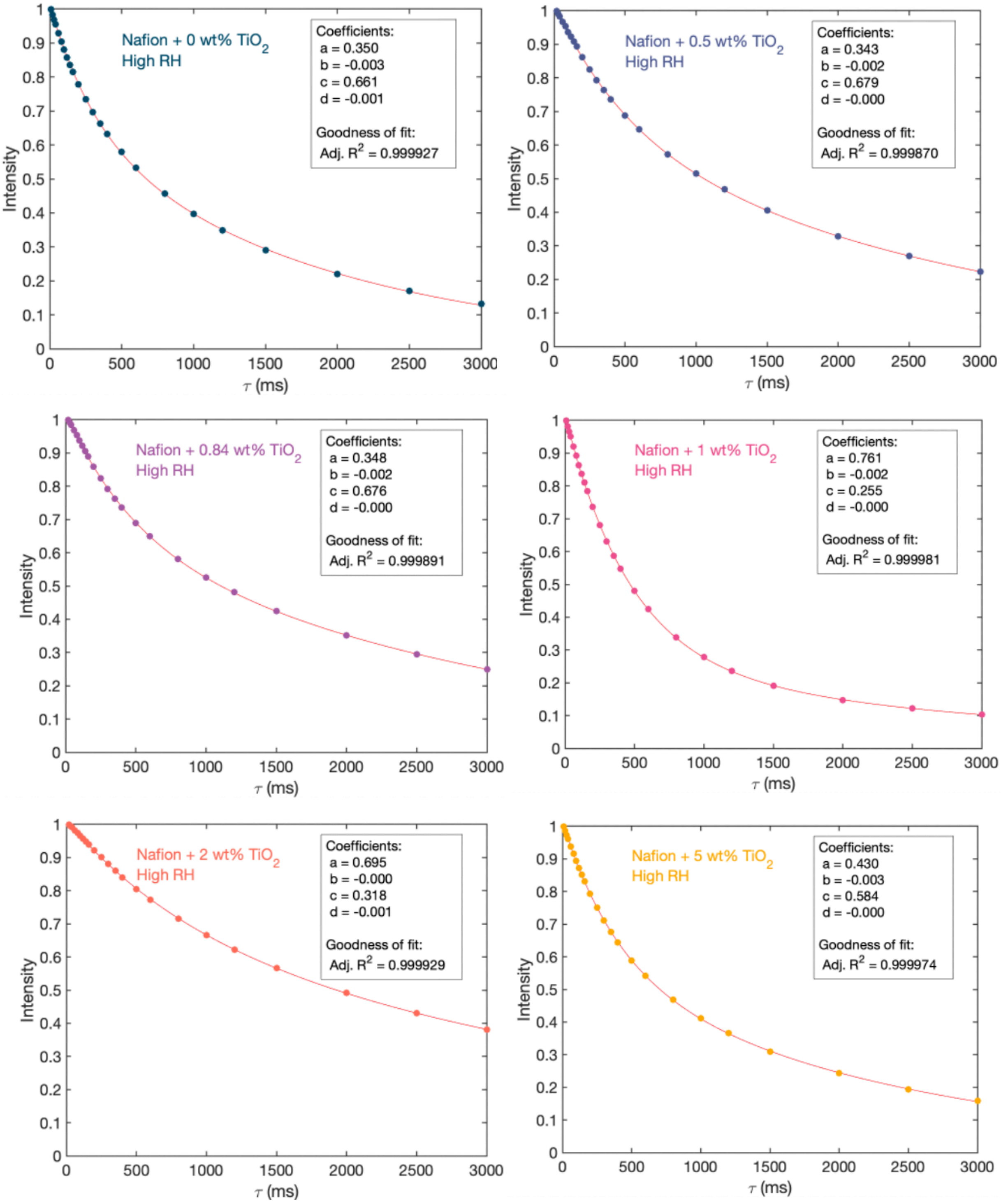

Figure S15: ^{1}H NMR T_2 decay fits for high relative humidity samples

References

(1) Amjadi, M.; Rowshanzamir, S.; Peighambardoust, S.; Hosseini, M.; Eikani, M. Investigation of physical properties and cell performance of Nafion/TiO2 nanocomposite membranes for high temperature PEM fuel cells. *International Journal of Hydrogen Energy* **2010**, *35*, 9252–9260.

(2) Bazrgar bajestani, M.; Mousavi, S. A. Effect of casting solvent on the characteristics of Nafion/TiO2 nanocomposite membranes for microbial fuel cell application. *International Journal of Hydrogen Energy* **2016**, *41*, 476–482.

(3) Jin, Y.; Qiao, S.; Zhang, L.; Xu, Z. P.; Smart, S.; da Costa, J. C. D.; Lu, G. Q. Novel Nafion composite membranes with mesoporous silica nanospheres as inorganic fillers. *Journal of Power Sources* **2008**, *185*, 664–669.

(4) Pineda-Delgado, J. L.; Gutierrez B, C. K.; Rivas, S.; Arjona, N.; Arriaga, L. G.; Chávez-Ramirez, A. U. Synthesis and evaluation of HfO2 as a prospective filler in inorganic–organic hybrid membranes based on Nafion for PEM fuel cells. *Nanotechnology* **2019**, *30*, 105707.

(5) Shao, Z.-G.; Xu, H.; Li, M.; Hsing, I.-M. Hybrid Nafion–inorganic oxides membrane doped with heteropolyacids for high temperature operation of proton exchange membrane fuel cell. *Solid State Ionics* **2006**, *177*, 779–785.

(6) Ercelik, M.; Ozden, A.; Devrim, Y.; Colpan, C. O. Investigation of Nafion based composite membranes on the performance of DMFCs. *International Journal of Hydrogen Energy* **2017**, *42*, 2658–2668.

(7) Ketpang, K.; Son, B.; Lee, D.; Shanmugam, S. Porous zirconium oxide nanotube modified Nafion composite membrane for polymer electrolyte membrane fuel cells operated under dry conditions. *Journal of Membrane Science* **2015**, *488*, 154–165.

(8) Kim, J.-H.; Kim, S.-K.; Nam, K.; Kim, D.-W. Composite proton conducting membranes based on Nafion and sulfonated SiO2 nanoparticles. *Journal of Membrane Science* **2012**, *415-416*, 696–701.

(9) Matos, B.; Isidoro, R.; Santiago, E.; Fonseca, F. Performance enhancement of direct ethanol fuel cell using Nafion composites with high volume fraction of titania. *Journal of Power Sources* **2014**, *268*, 706–711.

(10) Kim, D. J.; Jo, M. J.; Nam, S. Y. A review of polymer–nanocomposite electrolyte membranes for fuel cell application. *Journal of Industrial and Engineering Chemistry* **2015**, *21*, 36–52.

(11) Saccà, A.; Carbone, A.; Passalacqua, E.; D'Epifanio, A.; Licoccia, S.; Traversa, E.; Sala, E.; Traini, F.; Ornelas, R. Nafion–TiO2 hybrid membranes for medium temperature polymer electrolyte fuel cells (PEFCs). *Journal of Power Sources* **2005**, *152*, 16–21.

(12) Majsztrik, P. W.; Satterfield, M. B.; Bocarsly, A. B.; Benziger, J. B. Water sorption, desorption and transport in Nafion membranes. *Journal of Membrane Science* **2007**, *301*, 93–106.

(13) Motupally, S.; Becker, A. J.; Weidner, J. W. Diffusion of Water in Nafion 115 Membranes. *Journal of The Electrochemical Society* **2000**, *147*, 3171.

(14) Burnett, D. J.; Garcia, A. R.; Thielmann, F. Measuring moisture sorption and diffusion kinetics on proton exchange membranes using a gravimetric vapor sorption apparatus. *Journal of Power Sources* **2006**, *160*, 426–430.

(15) Takamatsu, T.; Hashiyama, M.; Eisenberg, A. Sorption phenomena in nafion membranes. *Journal of Applied Polymer Science* **1979**, *24*, 2199–2220.

(16) Ochi, S.; Kamishima, O.; Mizusaki, J.; Kawamura, J. Investigation of proton diffusion in Nafion®117 membrane by electrical conductivity and NMR. *Solid State Ionics* **2009**, *180*, 580–584.

(17) Zawodzinski, T. A. J.; Neeman, M.; Sillerud, L. O.; Gottesfeld, S. Determination of water diffusion coefficients in perfluorosulfonate ionomeric membranes. *The Journal of Physical Chemistry* **1991**, *95*, 6040–6044.

(18) Robert, M.; Kaddouri, A. E.; Perrin, J.-C.; Leclerc, S.; Lottin, O. Towards a NMR-Based Method for Characterizing the Degradation of Nafion XL Membranes for PEMFC. *Journal of The Electrochemical Society* **2018**, *165*, F3209.

(19) Ye, G.; Hayden, C. A.; Goward, G. R. Proton Dynamics of Nafion and Nafion/SiO2 Composites by Solid State NMR and Pulse Field Gradient NMR. *Macromolecules* **2007**, *40*, 1529–1537.

(20) Hammer, R.; Schönhoff, M.; Hansen, M. R. Comprehensive picture of water dynamics in nafion membranes at different levels of hydration. *The Journal of Physical Chemistry B* **2019**, *123*, 8313–8324.

(21) D'Epifanio, A.; Navarra, M. A.; Weise, F. C.; Mecheri, B.; Farrington, J.; Licoccia, S.; Greenbaum, S. Composite Nafion/Sulfated Zirconia Membranes: Effect of the Filler Surface Properties on Proton Transport Characteristics. *Chemistry of Materials* **2010**, *22*, 813–821.

(22) Yeo, S. C.; Eisenberg, A. Physical properties and supermolecular structure of perfluorinated ion-containing (nafion) polymers. *Journal of Applied Polymer Science* **1977**, *21*, 875–898.

(23) Balwani, A.; Davis, E. M. Anomalous, Multistage Liquid Water Diffusion and Ionomer Swelling Kinetics in Nafion and Nafion Nanocomposites. *ACS Applied Polymer Materials* **2020**, *2*, 40–54.

(24) Pivovar, A. M.; Pivovar, B. S. Dynamic Behavior of Water within a Polymer Electrolyte Fuel Cell Membrane at Low Hydration Levels. *The Journal of Physical Chemistry B* **2005**, *109*, 785–793.

(25) Perrin, J.-C.; Lyonnard, S.; Volino, F. Quasielastic Neutron Scattering Study of Water Dynamics in Hydrated Nafion Membranes. *The Journal of Physical Chemistry C* **2007**, *111*, 3393–3404.

(26) Chan, E. P.; Frieberg, B. R.; Ito, K.; Tarver, J.; Tyagi, M.; Zhang, W.; Coughlin, E. B.; Stafford, C. M.; Roy, A.; Rosenberg, S.; Soles, C. L. Insights into the Water Transport Mechanism in Polymeric Membranes from Neutron Scattering. *Macromolecules* **2020**, *53*, 1443–1450.

(27) Kusoglu, A.; Weber, A. Z. *Polymers for Energy Storage and Delivery: Polyelectrolytes for Batteries and Fuel Cells*; 2012; Chapter 11, pp 175–199.

(28) Zhao, Q.; Majsztrik, P.; Benziger, J. Diffusion and Interfacial Transport of Water in Nafion. *The Journal of Physical Chemistry B* **2011**, *115*, 2717–2727.
